# Multi-model genome-wide association analysis of agronomic traits in a Kabuli chickpea MAGIC-subset population (*Cicer arietinum* L.) across Mediterranean environments

**DOI:** 10.3389/fpls.2026.1860578

**Published:** 2026-07-21

**Authors:** Fatoumata Farida Traoré, Quahir Sohail, Achraf El Allali, Yasir Serag Alnor Gorafi, Kamal Hejjaoui, Aladdin Hamwieh, Tawffiq Instanbuli, Said Boughribil, Moez Amri

**Affiliations:** 1 Laboratory of Biotechnology, Agri-Food, Materials and Environment, Faculty of Science and Technology, University Hassan II of Casablanca, Mohammedia, Morocco; 2 College of Agriculture and Environmental Science (CAES), Mohammed VI Polytechnic University (UM6P), Ben Guerir, Morocco; 3 International Center for Agricultural Research in the Dry Areas (ICARDA), Rabat, Morocco; 4 Institute of Biotechnology and Genetic Engineering, The University of Agriculture Peshawar, Peshawar, Pakistan; 5 Bioinformatics Laboratory, College of Computing, Mohammed VI Polytechnic University, Ben Guerir, Morocco; 6 Graduate School of Agriculture, Kyoto University, Kyoto, Japan

**Keywords:** agronomics performance, chickpea (Cicer arietinum L.), grain yield, GWAS, marker-trait association (MTA), Mediterranean environments

## Abstract

Chickpea is one of the most consumed legumes due to its high nutritional value and accessibility to low-income populations. However, due to climate change, chickpea cultivation is exposed to various environmental stresses affecting its production and productivity. This study evaluates the agronomic performance of 168 MAGIC subset population across two Mediterranean environments, in Marchouch (Morocco) and Terbol (Lebanon). Genome-wide association studies (GWAS) were conducted to identify potential marker-trait associations (MTAs) using the general linear model (GLM), the mixed linear model (MLM), and the fixed- and random-effect circulant probability unification (FarmCPU), with kinship and principal components used as covariates. The results revealed high genetic variation among the genotypes tested, with significant genotype-by-environment interactions for most traits studied. Genotypes with good agronomic performance (M-1407, M-2038, M-2079, M-242, M-2551, and M-987) were identified under both environments. Early flowering and maturation resulted in a significant increase in grain yield of around 66%. Grain yield varied from 151.85 to 882.7 g m-2 and from 183.59 to 365.76 g m-2 under Marchouch and Terbol conditions, respectively, showing higher genetic variation under Marchouch than under Terbol. Correlation analysis revealed strong, significant correlations among the studied traits. GWAS revealed clear genetic variation across environments, with Marchouch showing stronger and more consistent association signals than Terbol. In total, 72 reliable MTAs were detected for phenological traits, 38 for plant height, 18 for grain yield, and 197 for hundred-seed weight across both sites. A major genomic hotspot on chromosome 4 (11.97–13.63 Mbp) harbored stable and pleiotropic MTAs. Functional annotation of regions surrounding significant SNPs revealed 59 putative candidate genes, highlighting potential biological processes related to growth, signaling, and stress responses that require further validation. These results provide a foundation for further research on marker-assisted selection to improve chickpea productivity and yield stability in stressed environments.

## 1 Introduction

Due to rising temperatures and extreme weather events, climate change can impact water resource availability and soil fertility, leading to decreased food production (Kadiyala et al., 2016), and increased food prices. In this regard, many researchers predict that drought, increased CO_2_ concentrations, and rising global temperatures will gradually increase in subtropical and semi-arid ecological zones (Wang et al., 2016). At the same time, population growth is driving increased demand for food, putting additional pressure on food systems. Currently, nearly 8 billion individuals inhabit our planet, and by 2050, this number is projected to exceed 9 billion (United Nations, 2022). Ensuring food security for this rapidly expanding population, both in terms of quantity and quality, poses a major challenge in the face of growing climate variability and resource scarcity.

Food legumes are of great importance for ensuring food security, especially in the current context of climate change and population growth. Chickpea (*Cicer arietinum* L.) is one of the most important legume crops, well adapted to arid and semi-arid conditions. It was first domesticated in the Middle East around 7450 years ago (Maiti and Wesche-Ebeling, 2001), from where it spread to the Levant and eventually to the whole Mediterranean basin. Currently, it is cultivated in an area of 14.09 Mha, with an annual production of 16.51 Mt (FAOSTAT, 2025), and ranks second among food legumes in global production. Due to its richness in proteins and essential mineral elements (Jukanti et al., 2012), chickpea is qualified as a complete food. Chickpea seeds are also characterized by a high content of assimilable carbohydrates (50%), a low fat content (6.48%), and a crude fiber content of 3.82% (Jukanti et al., 2012).

Over the past few decades, chickpea breeding programs have focused on developing and selecting genotypes that exhibit stable agronomic performance in different environments despite climate variations and environmental constraints (Srungarapu et al., 2022; Karimizadeh et al., 2023). Chickpea is traditionally grown in arid and semi-arid areas where it is subjected to major abiotic stresses, including drought and low and high temperatures (Karimizadeh et al., 2023). The occurrence of these stresses, particularly at flowering and during seed-filling, can affect their growth and yield (Prasad et al., 2017). The most important traits in plants, such as yield, are complex, controlled by many genes, and are highly influenced by the environment (Nunavath et al., 2020). Several studies have shown that the genotype × environment interaction (G × E) affects chickpeas agronomic parameters (Imtiaz et al., 2013). This interaction complicates the selection of superior genotypes in breeding programs, as it influences the expected responses and reduces the heritability of traits (Roorkiwal et al., 2018b; Karimizadeh et al., 2023). Analyzing the effects of this G × E interaction is essential for developing chickpea varieties adapted to specific environments, ensuring stable and sustainable production (Roorkiwal et al., 2018b). Therefore, for chickpea breeding, it is crucial to understand the G × E interaction, as well as the genetic basis underlying phenological traits, grain yield, and yield-related traits.

Genome-wide association studies (GWAS) based on linkage disequilibrium (LD) has become a powerful approach for identifying genomic regions controlling complex traits thereby facilitating the development of marker-assisted breeding (Flint-Garcia et al., 2003). The effectiveness of GWAS, largely depends on the extent of genetic diversity and recombination present within the population. Exploiting genetic diversity is therefore fundamental for dissecting quantitative traits and identifying quantitative trait loci (QTLs) in chickpea. To date, most genetic mapping studies targeting agronomic traits, yield components, and resistance to biotic and abiotic stresses have relied primarily on either biparental populations or diversity panels (Kujur et al., 2015a; Roorkiwal et al., 2018a; Barmukh et al., 2021; Nguyen et al., 2022). However, biparental populations are constrained by limited allelic diversity and relatively few recombination events, which restricts mapping resolution and reduces the precision of QTL localization. To overcome these limitations, multiparent advanced generation intercross (MAGIC) populations have emerged as powerful resources for investigating the genetic architecture of complex traits (Huang et al., 2015). Their multiple-founder design increases allelic diversity, enhances recombination frequency, and accelerates LD decay, thereby improving mapping resolution and facilitating the accumulation of favorable alleles associated with agronomic performance and adaptation to both biotic and abiotic stresses (Kover et al., 2009; Bajaj et al., 2016; Scott et al., 2020).

Several chickpea MAGIC populations have recently been developed to broaden the genetic base of the breeding germplasm and to facilitate the discovery of favorable/desirable alleles associated with key agronomic traits (Roorkiwal et al., 2020). For instance, the ICRISAT MAGIC population, derived from eight founder parents, generated extensive phenotypic variation and enabled the identification of superior lines across rainfed, irrigated, and heat-stress environments (Samineni et al., 2021). Subsequent studies exploited this population for high-resolution genetic mapping and genome-wide association analyses. Using whole-genome resequencing and multi-environment phenotyping, Thudi et al. (2023) identified numerous marker- trait associations and QTLs for flowering time, maturity, plant height, seed weight, biomass, and harvest index under drought stress condition. More recently, Akinlade et al. (2025) characterized the allelic diversity, population structure, and linkage disequilibrium patterns of the same ICRISAT MAGIC population and identified novel genomic regions associated with flowering time and plant height through GWAS and haplotype-based analyses. These studies highlight the value of chickpea MAGIC populations for dissecting complex traits and accelerating genetic improvement. Among these resources, the ICARDA MAGIC population, derived from 12 founder genotypes and comprising 3,053 recombinant inbred lines (RILs), is one of the largest and most genetically diverse multiparental populations currently available in chickpea, providing valuable genetic resources for genetic analysis and breeding.

Nevertheless, previous studies on chickpea MAGIC populations have mainly focused on population development, genetic characterization, and the genetic dissection of individual traits. Studies combining genotype × environment analyses and GWAS in Kabuli chickpea MAGIC populations under contrasting Mediterranean environments remain scarce. We therefore hypothesize that evaluating a highly recombined ICARDA MAGIC-Subset population across contrasting Mediterranean environments would enhance the detection of robust marker - trait associations and facilitate the identification of superior genotypes.

To address this gap, we evaluated the MAGIC-Subset population under two contrasting environments with the objectives of; (i) assessing the genetic variability of the population and the magnitude of genotype × environment interactions for grain yield and related traits, (ii) identifying superior high-yielding genotypes across environments, and (iii) characterizing population structure and identifying marker- trait associations for agronomic traits through genome-wide association analyses.

## 2 Materials and methods

### 2.1 Plant material and field experiments

A MAGIC-subset Kabuli chickpea collection of 168 F8 lines was used in this study. These lines were selected from a MAGIC population of 3053 Recombinant Inbred Lines (RILs) developed from multiple crosses using 12 different parents carrying the most interesting alleles for resistance to major biotic and abiotic stresses (**Supplementary Table 1**). These parents were selected to maximize genetic diversity and combine complementary alleles for adaptation to major chickpea production constraints. The list include elite cultivated chickpea germplasm and wild *Cicer reticulatum* accessions carrying valuable alleles for resistance to Ascochyta blight, Fusarium wilt, drought tolerance, heat adaptation, and yield performance. ICC4958 is a globally recognized donor for drought tolerance and root traits, while the wild accessions ILWC81 and ILWC292 broaden the genetic base and contribute novel alleles for resistance to biotic and abiotic stresses. The crosses were performed at ICARDA, Terbol research station in Lebanon. A preliminary evaluation of the phenological and agronomic performance of this MAGIC population was conducted at the ICARDA research stations in Marchouch (Morocco) and Terbol (Lebanon). Based on these evaluations, a representative subset of 168 lines was selected to capture broad phenotypic diversity while maintaining balanced representation across flowering-time classes (early, intermediate, and late), seed size and yield-potential categories (low, medium, and high). This strategy aimed to maximize phenotypic variability and ensure balanced representation of the diversity present in the original population, thereby reducing potential sampling bias in subsequent association analyses. The association panel, therefore, consisted of 168 MAGIC lines, additionally three check varieties (Ghab-2, Ghab-4, and Ghab-5) were included in the field experiment as repeated controls, resulting in a total of 171 genotypes represented in the field layout. Ghab-2, Ghab-4, and Ghab-5 were selected as control genotypes due to their well-documented agronomic traits and proven adaptability to challenging environments. Ghab 2, Ghab 4, and Ghab 5 were selected as control genotypes due to their well-documented agronomic traits and proven adaptability to challenging environments. Ghab 4 and Ghab 5, officially released in 2002, are recognized for their tolerance to water stress, whereas Ghab 2, introduced in 1986, exhibits notable resistance to cold and salinity., furthermore, extensive long term performance data for Ghab 2 is available across multiple locations, including Terbol and Marchouch, making it a reliable and well characterized standard check. These characteristics provide a robust comparative framework for assessing the performance of the studied genotypes (Malhotra et al., 2005; Nassif et al., 2005; Mazid et al., 2009).

**Figure 1:**
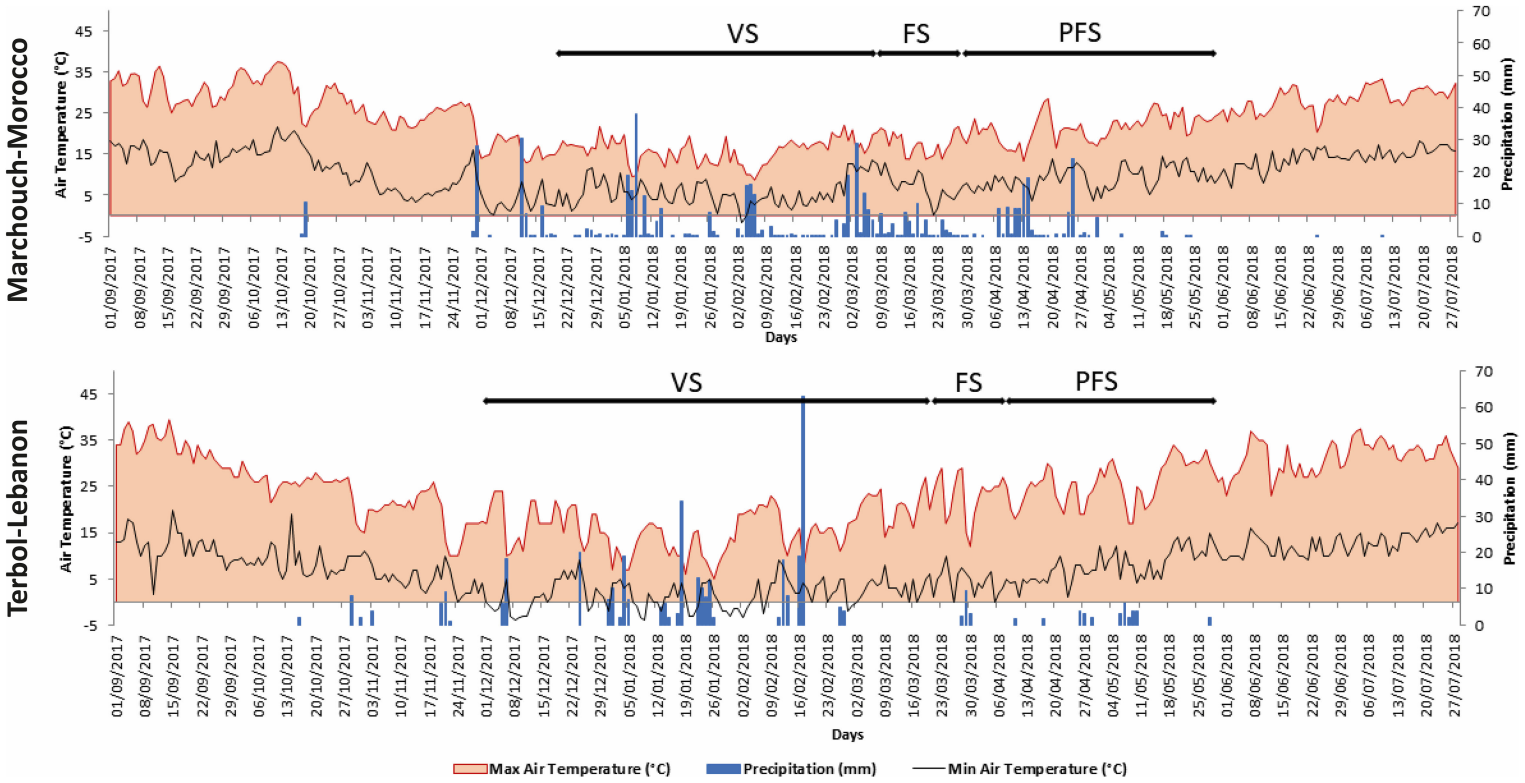
Precipitation (mm) and air temperature (°C) at Marchouch and Terbol during the cropping season 2017/2018. VS, Vegetative Stage; FS, Flowering Stage; PFS, Post Flowering Stage.

The field trials were conducted at two different locations: ICARDA research stations at Marchouch, Morocco (33.56 N, 6.63 W, 392 m) and Terbol, Lebanon (33.81 N, 35.99 W, 894 m) under semi-arid and warm Mediterranean climate conditions. The planting was conducted during the first (Marchouch) and third (Terbol) week of December 2017. Precipitations during the growing seasons were 504.6 mm and 384 mm at Marchouch and Terbol, respectively, and no irrigation was provided. The weather parameters for these two stations during the 2017/2018 crop year are presented in **Figure 1**. Field trials were conducted using an augmented design consisting of seven blocks. Owing to the large size of the MAGIC population, experimental lines were not replicated. Each block contained 24 entries, including three repeated check varieties, which were included across all blocks to allow adjustment for field heterogeneity and estimation of block-to-block variation. Each single plot consisted of 3 m rows with an inter-row spacing of 30 cm. Plant development was assessed throughout the crop year using the chickpea (*Cicer arietinum* L.) descriptor (International Board for Plant Genetic Resources, 1993). Before crop maturity, the number of days to flowering (D2F), number of days to maturity (D2M), and number of days from flowering to maturity (F2M) were recorded, while plant height (PH), grain yield (GY), and hundred seed weight (HSW) were recorded at crop maturity.

### 2.2 Statistical analysis of field data

Analysis of variance (ANOVA) and descriptive statistics were performed using the General Linear Model (GLM) procedure implemented in IBM SPSS Statistics 20.0 (IBM Corporation, Armonk, NY, USA). A combined ANOVA was used to assess the effects of genotype (G), environment (E) and the genotype × environment interaction (G×E).

Phenotypic data were subsequently analyzed separately for each environment using the R package *statgenSTA*. For each trait, a row-column linear mixed model accounting for block and spatial field variation was fitted using plot row and column coordinates. Genotype and block effects were treated as random factors, and best linear unbiased predictors (BLUPs) were estimated from the fitted models and subsequently used for downstream analyses. Variance components and broad-sense heritability estimates were extracted from the fitted mixed models. Summary data were then reported as ranges, means, and heritability values.

Pearson’s correlation coefficient was determined for the two environments using the *GGally* and *ggplot2* packages in RStudio. Principal component analysis (PCA) was performed using the *FactoMineR* and *Factoextra* packages in R (version 4.4.1) using standardized agronomic variables. Genotype grouping was obtained using k-means clustering applied to principal component coordinates.

### 2.3 Nucleic acids extraction and quantification

Three seeds from each MAGIC population line were cultivated under optimal conditions in the ICARDA greenhouse in Terbol. For nucleic acid extractions, three to four young leaflets were harvested from each line, snap frozen in liquid nitrogen, and stored at -80 °C for future use. Genomic DNA was extracted using the DNeasy^®^ 96 Plant Kit (QIAGEN, Hilden, Germany), and RNA was extracted using the RNeasy^®^ 96 Kit (QIAGEN) according to the manufacturer’s instructions. Tissue disruption was performed in a 96-well plate (QIAGEN Microtubes) using a Mixer Mill 300 (Retsch^®^, Haan, Germany), and eluted in 40 µL elution buffer, then stored at -80 °C. DNA and RNA were quantified using a Thermo Scientific™ NanoDropTM UV-Visible spectrophotometer, and their integrity was assessed with a TapeStation 2200 platform, utilizing either gDNA ScreenTape or RNA ScreenTape (Agilent Technologies, Santa Clara, CA, USA).

### 2.4 SNP genotyping and filtering for GWAS

The genotyping workflow consisted of two sequential steps. First, transcriptome-based genotyping-by-sequencing (GBS-t) was used for genome-wide SNP discovery and target enrichment assay design. Subsequently, to genotype the MAGIC population, capture-based sequencing of genomic DNA was performed using the predefined SNP panel. Supplementary for genotyping of the MAGIC population.

#### 2.4.1 Genotyping by sequencing with transcriptomics

Library preparation for GBS-t was performed according to the methods described by Sudheesh et al. (2021). After RNA extraction from the lines, paired-end sequencing libraries were constructed with 350 bp insert sizes using the SureSelect Strand Specific RNA Library Preparation Kit (Agilent Technologies) following the manufacturer’s instructions. The quality of the obtained libraries was assessed using the TapeStation 2200 platform (Agilent Technologies). Equal quantities of each library with a unique barcode were then pooled to create a single sample suitable for next-generation sequencing technology. The pooled libraries were quantified using the KAPA library quantification kit (KAPA Biosystems, Wilmington, MA, USA).

Sequencing was performed using an Illumina HiSeq 2000 sequencing platform producing 2 × 150 bp reads. Each sample was sequenced to a depth of 5 to 6 million read pairs (Illumina Inc., San Diego, CA, USA). The sequencing data fastq files were filtered using a custom perl script to remove adapter sequences as well as low-quality reads and bases (Q ≤ 30). Reads with three consecutive unassigned nucleotides (N) were also eliminated. Additionally, all reads shorter than 50 bp were removed from the final dataset. Because GBS-t was based on RNA-derived sequencing libraries, high-quality reads were aligned to the CDC Frontier chickpea reference genome (Varshney et al., 2013) using the splice-aware RNA-seq aligner TopHat2 (version 2.1.1), following the protocol of Sudheesh et al. (2021). SNP polymorphisms were identified using SAMtools and BCFtools, which generated the Variant Call Format (VCF) file. The VCF file was filtered based on various parameters, including depth (DP → 10), minor allele frequency (MAF 0.05), maximum missing data (20%), and base quality (Q30) using vcftools. For the design of target enrichment arrays, a final set of SNPs was selected based on uniform distribution across the genome, and flanking sequences of 100 bp upstream and downstream of each target SNP were sent to Agilent Technologies for target capture array design.

#### 2.4.2 Capture-based sequencing

The target enrichment assay developed from the SNPs identified during the GBS-t discovery phase was subsequently used to genotype the MAGIC population. Target enrichment was performed according to the manufacturer’s instructions (SureSelect Target Enrichment [Agilent Technologies]). High-quality DNA (200 ng) was subjected to random fragmentation using the MspJI restriction enzyme (New England Biolabs, Ipswich, MA, USA) (Shinozuka et al., 2015). All reads were sequenced in pairs on the MiSeq platform (Illumina Inc), producing approximately 2 to 3 million read pairs per line. The obtained sequence data were trimmed as before and then aligned to the reference genome sequence of the chickpea, CDC Frontier (Varshney et al., 2013), using BWA MEM. SNP calling was performed using SAMtools and BCFtools, based on the predefined SNP targets included in the target enrichment assay. The VCF output was processed with the same parameters as those used for filtering GBS-t sequencing data using vcftools. Linkage disequilibrium (LD) pruning was performed in PLINK v1.9 to minimize marker redundancy arising from high SNP correlation (r² > 0.9). This filtering reduced the dataset from 765,803 to 24,675 SNPs, ensuring a set of largely independent markers for subsequent analyses (Akinlade et al., 2025). In addition, a schematic overview of the genotyping workflow, including GBS-t-based SNP discovery, target enrichment assay design, capture-based sequencing, SNP filtering, and LD pruning, is summarized in the **Supplementary Figure 1** to improve clarity and reproducibility.

### 2.5 Analysis of population structure and linkage disequilibrium

Population structure was evaluated using multiple complementary approaches based on genome-wide SNP marker information. Population structure was inferred using the R package LEA through sparse non-negative matrix factorization (sNMF). The *snmf* function was applied to estimate individual admixture coefficients from the genotypic matrix and to generate least squares estimates of ancestry proportions (Frichot et al., 2014). An entropy criterion was used to determine the optimal number of ancestral populations (K). The number of genetic clusters (K = 1 to 15) was tested using ten independent runs per K value to ensure convergence and consistency of results. In addition to sNMF, discriminant analysis of principal components (DAPC) (Jombart et al., 2010), implemented in the R package adegenet, was conducted to further characterize the genetic structure and identify groups of individuals sharing common ancestry. The analysis was performed without prior information about population origin, testing K values ranging from 1 to 10. The *find.clusters* function was used to determine the optimal number of clusters based on the Bayesian Information Criterion (BIC). The number of principal components (PCs) retained in the DAPC was optimized using the *xvalDAPC* function. Genetic clustering results were visualized using membership bar plots and scatter plots of discriminant functions.

Genome-wide linkage disequilibrium was calculated in TASSEL using pairwise r² values among SNPs across the eight chickpea chromosomes. LD decay was then visualized in R4.4.1) by plotting the average r² values against increasing physical distance (bp), following the procedure described by Remington et al (Remington et al., 2001).

A Neighbor-Joining tree was constructed based on a shared-allele dissimilarity matrix derived from the SNP dataset, following Perrier and Flori (Perrier et al., 2003), to assess genetic relationships among accessions.

### 2.6 Genome-wide association study

GWAS was performed on six agronomic traits evaluated across the two contrasting environments, Marchouch and Terbol, using a total of 24,675 SNP markers. Analyses were conducted with the R package mMVP^1^. Six statistical models were tested to determine the most appropriate approach: GLM (K), GLM (K + PCA), MLM (K), MLM (K + PCA), FarmCPU (K), and FarmCPU (K + PCA). These models incorporate kinship (K) and/or principal components (PCA) to account for population structure and genetic relatedness, thereby minimizing false positive associations. The best GWAS model was chosen based on the comparison between the observed and expected p-values in quantile–quantile (QQ) plots. Among all models, FarmCPU (K + PCA) provided the best fit. The significant SNPs associated with the studied traits were selected based on an unadjusted p-value threshold of 10^-^³ (–log_10_P ≥ 3.0). We used an unadjusted p-value to declare significance because Bonferroni and FDR tests are considered far too conservative for association studies involving thousands of candidate variants at very low minor allele frequencies (Atri et al., 2019). Visualization of significant SNPs was done using Manhattan plots.

The candidate genes corresponding to significantly associated MTAs were searched for in the chickpea genome using the exact position of the significant SNP (Varshney et al., 2013). The biological functions of these candidate genes were determined using the following databases: Pulse Crops^2^ and UniProt Knowledge^3^.

## 3 Results

### 3.1 Germplasm evaluation and screening for agronomic performance under different environments

The two experimental sites exhibited contrasting climatic conditions significantly influenced agronomic performance of the chickpea tested genotypes. The phenotypic distributions of the measured traits are shown in **Supplementary Figure 2**. At Marchouch (Morocco), higher rainfall and moderate temperatures (0 to 33.4 °C) led to earlier flowering, a 15-day longer grain-filling period, and overall higher productivity. In contrast, Terbol (Lebanon) experienced harsher conditions, including prolonged drought and extreme temperatures (-4 to 37.5 °C) during germination and reproductive stages, causing delayed flowering (by ~32 days), reduced plant height, and lower yields.

Analysis of variance revealed highly significant differences (p < 0.01) among genotypes for all agronomic parameters (**Table 1**). The effect of the environment was significant for all traits except HSW. Similarly, a significant G × E interaction was observed for D2M, PH, and GY.

**Table 1:** Mean squares of combined analysis of variance for agronomic parameters at Marchouch and Terbol.

Source	D2F	D2M	F2M	PH	GY	HSW
Environment (E)	1492.34^**^	3441.56^**^	174.06^**^	1838.78^**^	103.44^**^	3.09ns
Genotype (G)	4.46^**^	9.97^**^	3.04^**^	2.86^**^	2.32^**^	3.01^**^
G * E	1.53ns	5.34^**^	1.59ns	1.81^*^	1.80^*^	0.63ns

Significant difference at: ^*^ p < 0.05, ^**^ p < 0.01, ns denotes a non-significant difference.

D2M exhibited high heritability, with values of 0.88 and 0.82 in Marchouch and Terbol, respectively. For the other parameters, heritability values varied across environments (**Table 2**). Moderate and high heritability values were observed for D2F (0.39 and 0.91), F2M (0.49 and 0.72), and HSW (0.32 and 0.69) in both stations. On the other hand, GY showed low and moderate heritability (0.28 and 0.55, respectively, in Marchouch and Terbol),. This variability in heritability suggests that the relative contributions of genetic and environmental factors to phenotypic variation differed between environments.

**Table 2:** Range, mean value and heritability (h^2^) of agronomic parameters at Marchouch and Terbol.

	Marchouch				Terbol			
Trait	Min	Max	Mean	h 2	Min	Max	Mean	h2
D2F	73.72	98.34	87.28	0.39	105.35	131.52	119.05	0.91
D2M	146.489	181.22	159.08	0.88	168.78	185.17	176.25	0.82
F2M	56.01	95.58	72.40	0.49	46.42	68.86	57.29	0.72
PH (cm)	54.98	88.41	74.75	0.53	38.89	55.42	44.76	0.41
GY (g.m-2)	151.85	882.7	391.54	0.55	183.59	365.76	259.32	0.28
HSW (g)	20.60	33.44	25.29	0.32	18.63	43.87	26.51	0.69

The earliest flowering genotypes were M-1695 (Marchouch) and M-2551 (Terbol). Grain yield was on average 66% higher at Marchouch than at Terbol. However, higher genotypic variation in GY was observed at Marchouch (151.85 to 882.7 g m^-2^) compared to Terbol (183.59 to 365.76 g m^-2^). The highest GY was recorded for genotypes M-962 and M-499 at Marchouch and Terbol, respectively. Several tested lines outperformed the control genotypes. In Marchouch, Ghab 4 showed the highest check yield (302.88 g·m^-^²), while in Terbol, Ghab 4 and Ghab 2 yielded 284.89 and 242.02 g·m^-^², respectively. However, the top performing MAGIC lines surpassed these controls, especially under the favorable conditions in Marchouch. Regarding seed weight (HSW), genotypic differences were more pronounced in Terbol, ranging from 18.63 g (M-1006) to 43.87 g (M-63), compared with 20.6 (M-1734) –33.44 g (M-2575) in Marchouch. These results underscore the influence of the environment on phenology and yield, and the potential of early-flowering genotypes for breeding climate-resilient chickpea lines.

**Figure 2:**
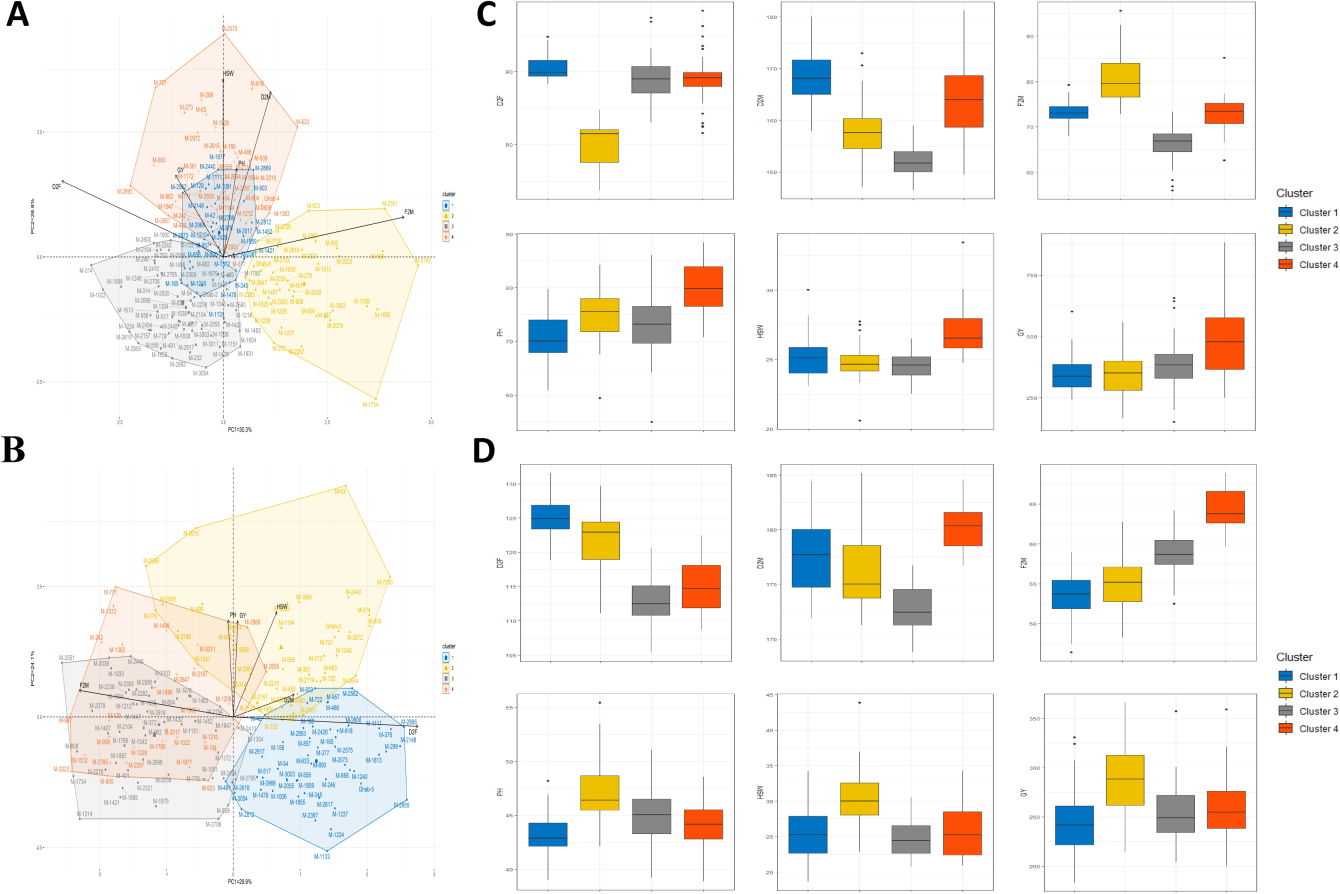
Principal component analysis (PCA) of agronomic traits and genotype clustering under Marchouch and Terbol environments. PCA biplots based on the first two principal components are shown for Marchouch **(A)** and Terbol **(B)**, illustrating the relationships among agronomic traits and genotype distribution patterns. Boxplots showing the distribution of agronomic traits across the identified PCA-derived clusters are presented for Marchouch **(C)** and Terbol **(D)**.

Principal component analysis (PCA) was performed on the different agronomic parameters studied. Under Marchouch conditions, the first two principal components PC1 (30.3%) and PC2 (26.8%) explained 57.1% of the total variation (**Figure 2A**). PC1 demonstrated a strong positive contribution of F2M (r = 0.96) against a negative association with D2F (r = -0.86), whereas PC2 was positively correlated with HSW (r = 0.79) and D2M (r = 0.73) (**Table 3**). Under Terbol conditions, the first two components PC1 (29.9%) and PC2 (24.1%) explained 54% of the total variability (**Figure 2B**). PC1 showed a high positive correlation with D2F (r = 0.98) and a negative correlation with F2M (r= -0.82), whereas PC2 had a high positive correlation with HSW (r = 0.71), PH (r = 0.66), and GY (r = 0.65) (**Table 3**). In both experimental locations, the PCA biplot (**Figure 2**) showed that all the tested genotypes could be grouped into four different clusters with 31, 37, 64, and 39 genotypes in each group at Marchouch, and 51, 41, 50, and 29 genotypes in each group at Terbol.

**Table 3:** Correlation matrix between the studied traits and the principal components PC1, PC2, and PC3 of the principal component analysis (PCA).

	Marchouch			Terbol		
Trait	PC 1	PC 2	PC 3	PC 1	PC 2	PC 3
D2F	**-0.865**	0.339	-0.297	**0.982**	-0.064	0.123
D2M	0.255	**0.737**	**-0.548**	0.323	0.152	**0.890**
F2M	**0.967**	0.178	-0.100	**-0.820**	0.181	**0.525**
PH	0.071	0.395	**0.754**	-0.028	**0.661**	**-0.462**
GY	-0.256	0.365	0.287	0.023	**0.657**	0.096
HSW	-0.002	**0.795**	0.151	0.231	**0.718**	0.027

The table shows the correlation coefficients of each trait with the three principal components, highlighting their contribution to genotype clustering. Bold values indicate the traits with the highest positive or negative correlations with each principal component, representing the variables that contribute most strongly to respective PCs.

At Marchouch, cluster 1 included late flowering genotypes with moderate HSW and GY. While cluster 2 consisted of early-flowering genotypes with a long grain-filling period (F2M) and low GY. Cluster 3 included late flowering genotypes with low GY and HSW. The fourth cluster grouped late flowering genotypes with high yield and large seeds (high HSW) (**Figure 2C**). In contrast, under Terbol conditions, the first cluster grouped the late flowering genotypes with short F2M, and low GY and HSW. Cluster 2 was mostly composed of genotypes with high GY, HSW, and PH. The third cluster contained early flowering genotypes with low GY and HSW. Finally, cluster 4 grouped early-flowering genotypes with a long grain-filling period (F2M) and moderate GY and HSW (**Figure 2D**).

**Figure 3:**
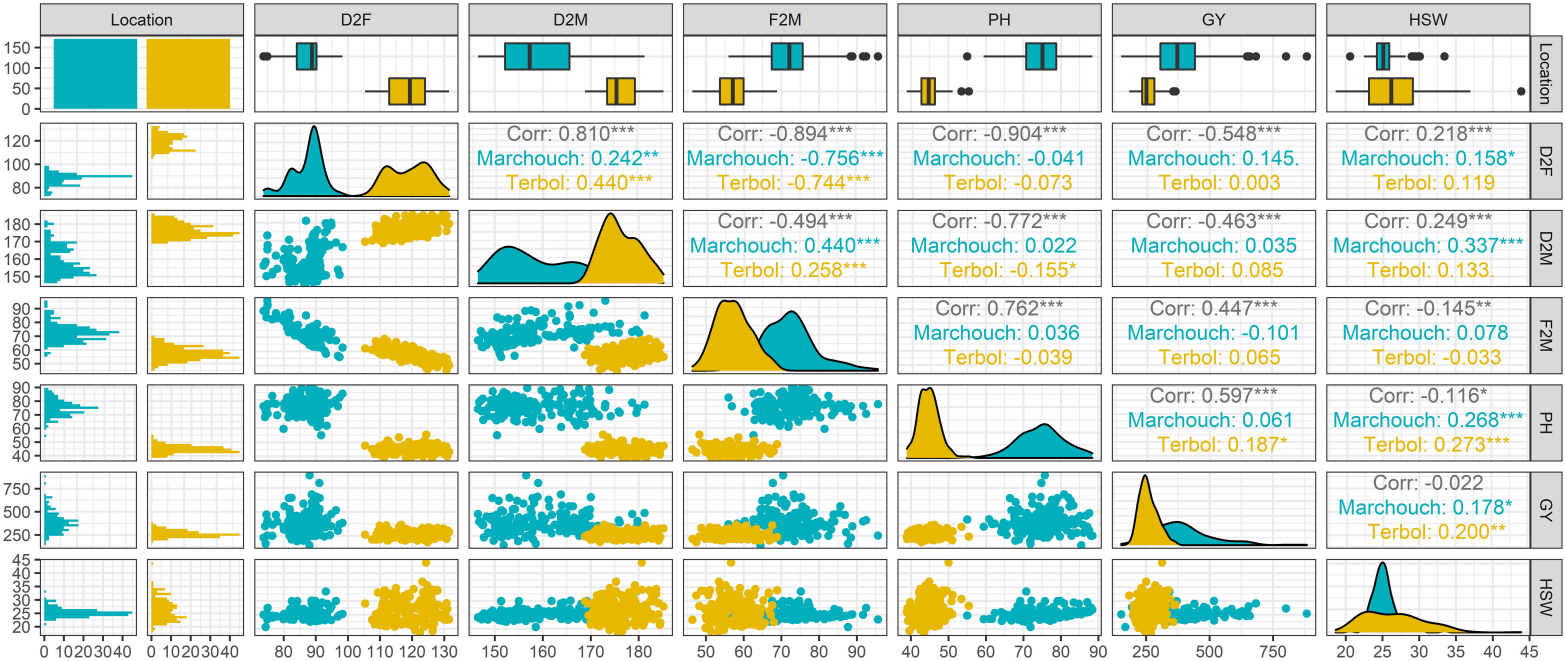
Pairwise correlations between the agronomic parameters recorded for all chickpea-tested genotypes at Marchouch and Terbol.

Pairwise correlation analysis of agronomic traits at both sites using aggregated data from two environments (**Figure 3**) revealed positive correlations between D2F and D2M (r = 0.81^***)^ and HSW (r = 0.22^***^). In contrast, it was negatively correlated with F2M (r = -0.89^***^), PH (r= -0.9^***^) and GY (r= -0.55^***^). GY was positively correlated with F2M (r = 0.45^***)^ and PH (r = 0.60^***^). Similarly, F2M was positively correlated with PH (r= 0.76^***^).

**Figure 4:**
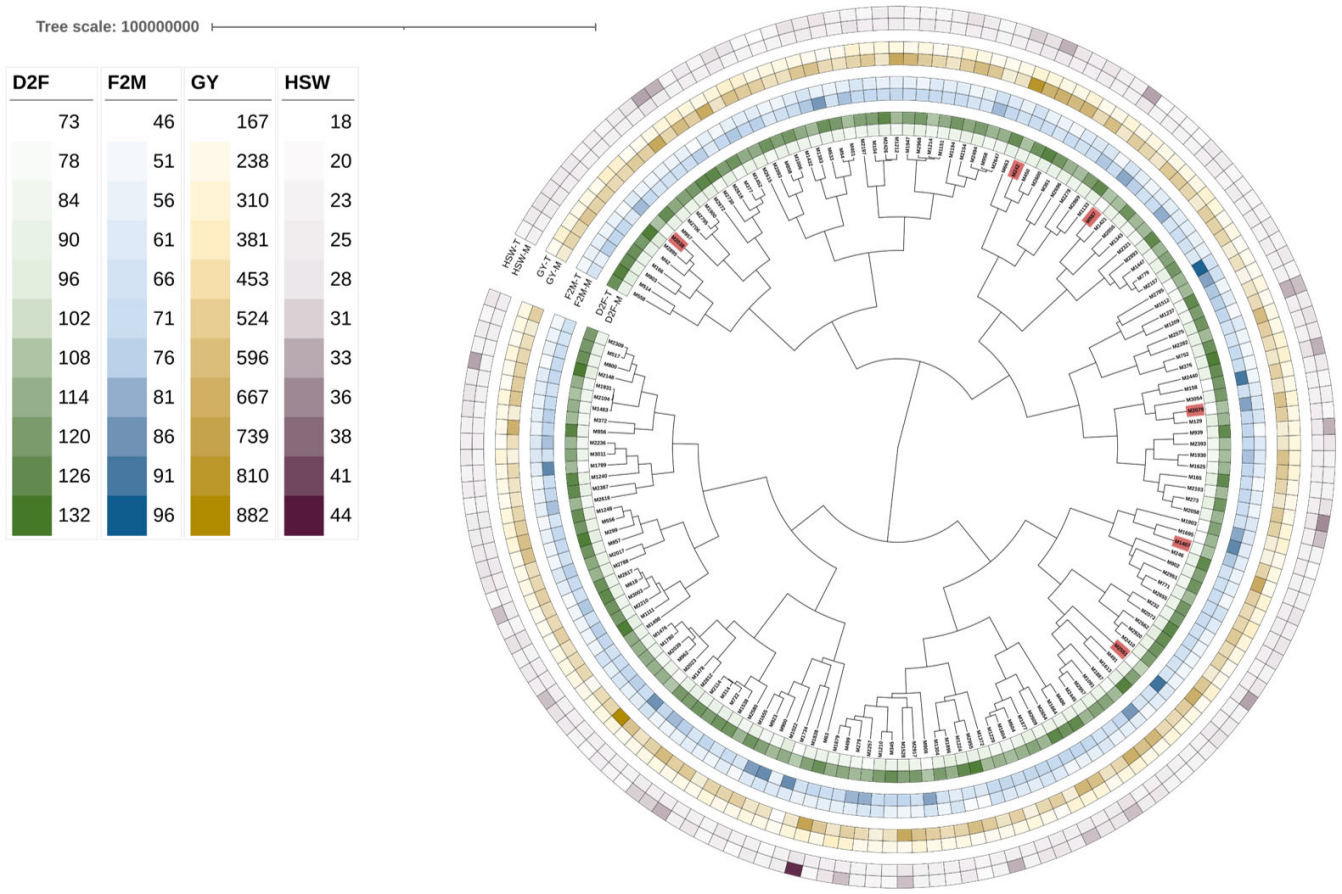
Dendogram showing the agronomic performances of the genotypes of this population at Marchouch (M) and Terbol (T). Genotypes highlighted in red: Genotypes with good agronomic performance in both environments.

The agronomic performance of all the tested genotypes under both environments revealed that most of the tested genotypes flowered comparatively late at Terbol (**Figure 4**). Under both environments, 85 genotypes showed a relatively high GY (> 250 gm^-2^) out of which 11 genotypes were early and only one had large seed size; genotype “M-63” showed HSW of more than 40 g (only at Terbol). To identify superior genotypes, grain yield, flowering time, and seed size were jointly considered across both environments. Genotypes exhibiting consistently high grain yield, relatively early flowering, and acceptable seed size were considered to have superior agronomic performance. Based on these combined criteria, six genotypes (M-1407, M-2038, M-2079, M-242, M-2551, and M-987) were identified as the most promising across both environments.

### 3.2 Population structure and linkage disequilibrium

**Figure 5:**
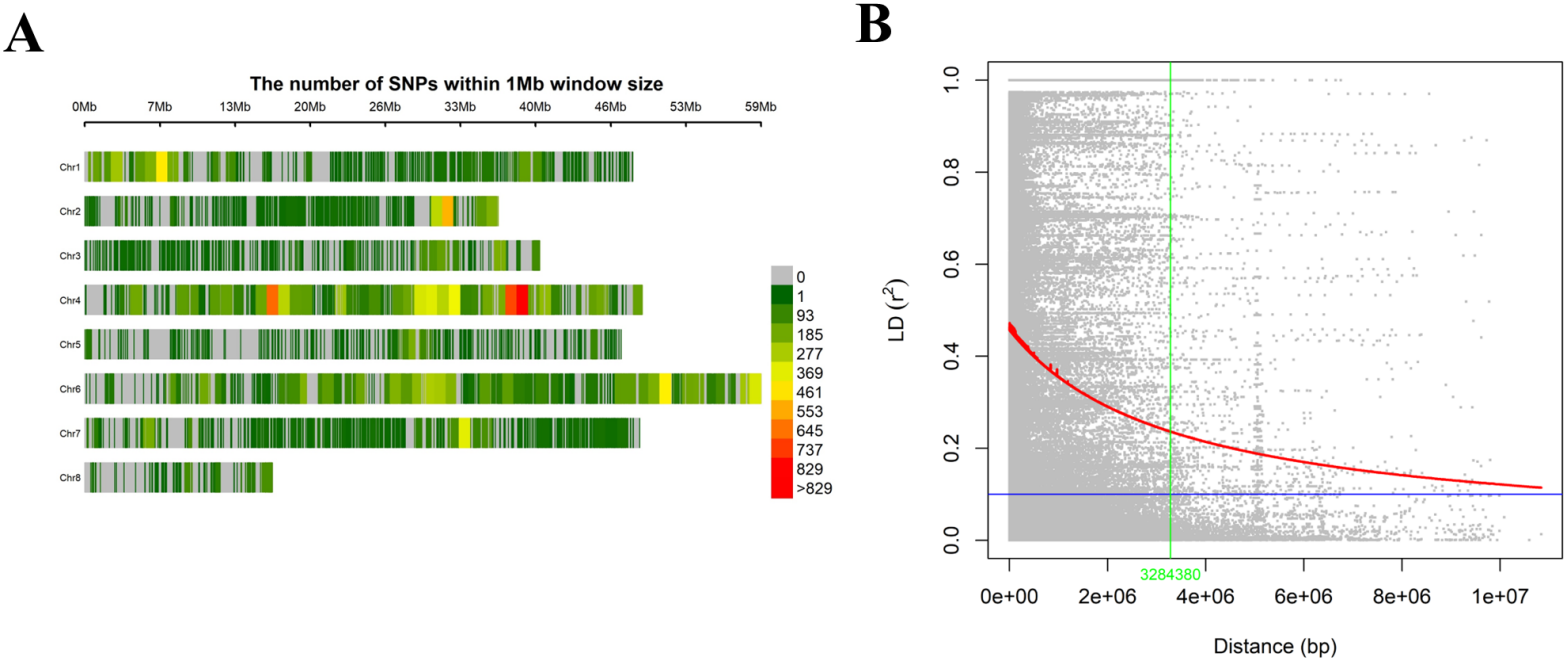
**(A)** SNP density plot showing the distribution of 24,675 SNPs across the eight chickpea chromosomes, expressed as the number of SNPs within 1 Mb genomic windows. **(B)** Genome-wide linkage disequilibrium (LD) decay plot illustrating the relationship between linkage disequilibrium (*r*^2^) and the physical distance between SNP marker pairs (bp).

The population structure and linkage disequilibrium of the studied genotypes were assessed using 24,675 high-quality SNPs (**Figure 5A**). Genome-wide LD analysis revealed a relatively high initial LD (r² ≈ 0.45), which declined gradually to r² ≈ 0.1 at approximately 3.3 Mb, indicating the presence of extended haplotype blocks typical of self-pollinating species (**Figure 5B**).

Population structure inferred using the LEA package identified K = 5 as the optimal number of ancestral groups based on the cross-entropy criterion (**Supplementary Figure 3A**). In addition, a DAPC analysis was performed without prior population information, yielding K values from 5 to 7, with K = 5 selected as the most biologically consistent model (**Supplementary Figure 3B**). The five genetic subpopulations comprised 29, 31, 26, 32, and 47 genotypes, respectively, reflecting uneven group sizes but a clear and coherent clustering pattern. A Neighbor-Joining phylogenetic tree was constructed from a shared-allele dissimilarity matrix derived from the SNP dataset (**Supplementary Figure 3C**). The tree grouped the accessions into five major clades, consistent with the five subpopulations identified by LEA and DAPC.

### 3.3 Genome-wide association studies for agronomic traits

**Figure 6:**
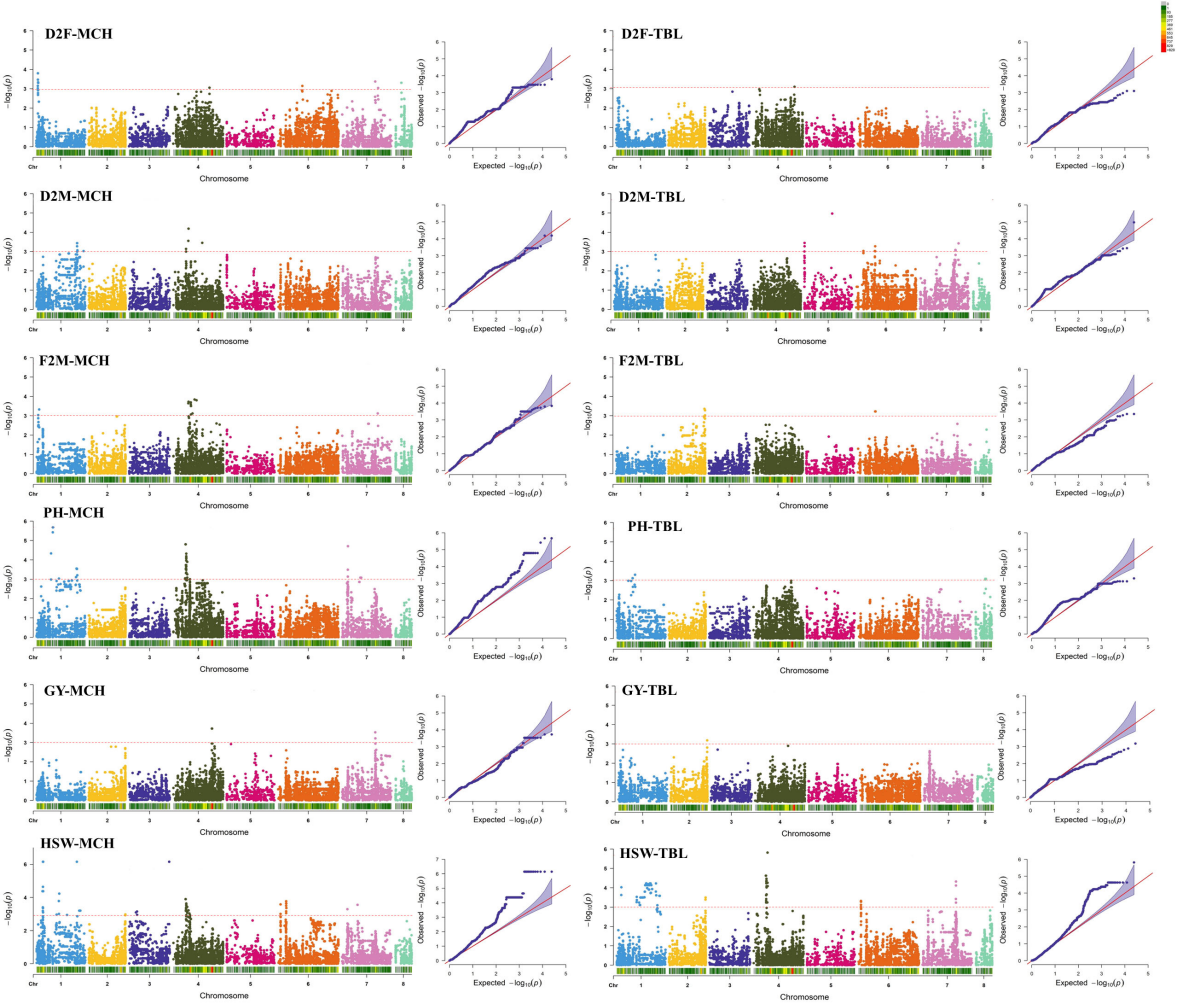
Manhattan and quantile-quantile (QQ) plots showing genome-wide association study (GWAS) results for agronomic traits under Marchouch (MCH) and Terbol (TBL) environments using the FarmCPU model with K + PCA as covariates. In the Manhattan plots, the red dashed line indicates the significance threshold for marker trait associations. QQ plots depict the comparison between observed and expected –log_10_(*P*) values. Each panel represents a different trait, including days to flowering (D2F), days to maturity (D2M), flowering-to-maturity duration (F2M), plant height (PH), grain yield (GY), and hundred-seed weight (HSW).

A genome-wide association study (GWAS) was conducted to identify marker–trait associations (MTAs) for agronomic traits evaluated at Marchouch and Terbol. Several statistical models were tested to determine the best-fitting approach. Model performance was evaluated by comparing observed and expected p-values in quantile–quantile (QQ) plots. Among all models, the FarmCPU (K + PCA) model provided the best fit. Manhattan plots displaying the significant MTAs for each trait based on the selected model are shown in **Figure 6** and **Supplementary Figures 4**-**7**. The results from the FarmCPU (K + PCA) model were compared with those from the five other models (**Supplementary Figures 3**-**6**) to identify the most reliable MTAs, defined as those consistently identified by at least three methods (**Table 4**). A significance threshold of –log10 (P) ≥ 3.0 was applied, leading to the detection of MTAs for agronomic traits at Marchouch and Terbol.

**Table 4:** Number of significant markers–trait associations detected across six genome-wide association study models for agronomic traits at Marchouch and Terbol, based on a -log_10_(p) threshold ≥ 3.

	Marchouch				Terbol			
Trait	Env	FarmCPU (K+PCA)	GLM (K+PCA)	MLM (K+PCA)	FarmCPU (K)	GLM (K)	MLM (K)	Reliable MTAs*
D2F	MCH	56	56	1	464	464	2	48
	TBL	2	2	0	584	584	0	2
D2M	MCH	20	20	3	119	119	2	5
	TBL	24	24	1	25	25	23	9
F2M	MCH	35	35	6	293	293	6	8
	TBL	6	6	5	645	645	1	5
PH	MCH	94	94	3	71	71	4	30
	TBL	8	8	0	1	1	0	0
GY	MCH	17	17	17	17	17	15	17
	TBL	1	1	0	2	2	1	1
HSW	MCH	213	213	94	11	784	161	168
	TBL	151	151	16	2	279	49	82

MTAs, marker-trait associations; Env, Environment; MCH, Marchouch; TBL, Terbol; Reliable MTAs *Marker–trait associations validated by two additional models, with FarmCPU (K + PCA) as reference.

A total of 56 SNPs were significantly associated with D2F at Marchouch, of which 48 were considered reliable. These associations were mainly located on chromosomes 1, 4, 6, 7, and 8. At Terbol, only two MTAs were detected on chromosome 4, suggesting a strong environmental influence on flowering time. For D2M, 20 MTAs (5 reliable) were detected at Marchouch and 24 (9 reliable) at Terbol. These MTAs were located on chromosomes 1 and 4 at Marchouch and on chromosomes 5, 6, and 7 at Terbol. Regarding F2M, 35 MTAs (8 reliable) were identified at Marchouch on chromosomes 1, 4, and 7, and 6 MTAs (5 reliable) at Terbol, located on chromosomes 2 and 6.

For PH, 94 MTAs (30 reliable) were detected at Marchouch, located on chromosomes 1, 4, and 7. In contrast, only eight associations were identified at Terbol, mainly on chromosomes 1 and 8. In total, 17 reliable MTAs were detected for GY at Marchouch, observed on chromosomes 4 and 7 against only one association was observed at Terbol on chromosome 2 (35.92 Mb). Hundred-seed weight (HSW) exhibited the largest number of significant associations, with 213 MTAs (168 reliable) identified at Marchouch, and 151 MTAs (82 reliable) detected at Terbol. At Marchouch, these MTAs were distributed across chromosomes 1, 3, 4, 6, and 7, while at Terbol, they were located on chromosomes 1, 2, 4, 6, and 7. A major association hotspot was consistently observed on chromosome 4, spanning 11.97–13.63 Mb, which was shared across traits and both environments. Notably, 57 MTAs for HSW were stable across the two sites, predominantly clustered within this region, highlighting a key genomic interval with potential broad environmental relevance for seed weight.

At Marchouch, a total of 68 pleiotropic loci were identified, including nine shared between D2F and F2M on chromosome 1 (1.79–1.84 Mb), 56 shared between HSW and PH on chromosome 1 (22.15 Mb) and chromosome 4 (11.97–13.66 Mb), one shared between D2M and F2M on chromosome 4 (14.66 Mb), and two loci associated with D2M, F2M, and HSW on chromosome 4 (14.88–14.89 Mb). No pleiotropic loci were detected at Terbol, highlighting the strong environmental specificity of genetic control at this site.

Overall, Marchouch showed broader and more consistent chromosomal signals, with Chr 4 emerging as a key hotspot controlling phenological and yield-related traits. In contrast, Terbol exhibited fewer and environment-specific associations, reflecting strong genotype × environment interactions.

Our results revealed 59 candidate genes linked to significant SNP markers underlying agronomic traits (**Supplementary Table 2**). These genes encode proteins with diverse annotated functions, including protein kinases, prolyl 4-hydroxylase, ascorbate ferrireductase, trehalose-phosphate phosphatase I, SAR DEFICIENT 4, and calmodulin-binding transcription activator 3. The functional annotations of these genes suggest potential involvement in biological processes related to growth, metabolism, stress responses, and signaling pathways. Nevertheless, the associations reported here are based on positional proximity to significant SNPs and should be considered as candidate hypotheses pending further validation.

## 4 Discussion

### 4.1 Agronomic performance and genetic variability

Improving stress resistance and increasing grain yield remain the major objectives of national and international chickpea breeding programs. In this study, a subset of chickpea genotypes selected from a MAGIC population was subjected to agronomic performance evaluation and genotypic variation assessment across two Mediterranean environments at Marchouch, Morocco, and Terbol, Lebanon. The results showed large genotypic variation and revealed significant differences among the genotypes studied for agronomic traits. A high level of heritability was recorded for D2M at Marchouch and for D2F and D2M at Terbol. Similar results have been reported in previous work in chickpeas for D2F (Khan et al., 2011) and D2M (Saxena and Nandedkar, 2021). A significant genotype × environment interaction (G × E) was also observed for D2M, PH, and GY. Despite these interactions, genotypes M-1407, M-2038, M-2079, M-242, M-2551 and M-987 exhibited relatively favorable agronomic performance across both environments, combining high grain yield, flowering earliness, and desirable plant architecture. These genotypes represent promising breeding lines that warrant further evaluation across additional environments and growing seasons to assess their broader adaptation and yield stability.

Under Marchouch conditions, most of the tested genotypes showed earlier flowering and a longer seed-filling duration than in the Terbol environment. Low temperatures during the vegetative stage disrupt the germination and delay chickpea plants emergence, extending their flowering and maturity dates (Arriagada et al., 2022). Therefore, it is likely that the observed delay in flowering among the genotypes in Terbol is due to the low temperatures (< 0 °C) experienced during their vegetative stage. Such delayed flowering may increase exposure to terminal drought and heat stress during reproductive stages, thereby reducing grain-filling efficiency. Under drought conditions, earlier flowering and maturing allow the chickpea plant to escape water stress at the end of the development cycle (Malhotra et al., 2004). Early flowering also positively affects grain yield by prolonging the reproductive phase and leading to a longer seed-filling period (F2M) (Kumar and Abbo, 2001; Sundaram et al., 2019). In the present study, the significant negative correlation between D2F and F2M across environments indicates that early-flowering genotypes tended to maintain a longer flowering-to-maturity period. In turn, the positive correlation between F2M and grain yield (GY) suggests that an extended reproductive phase may promote assimilate accumulation and seed development, thereby contributing to yield formation. Similar relationships have also been reported in chickpeas where longer reproductive period was associated with improved yield performance, particularly under mediterranean and terminal drought environments (Silim et al., 2009). Comparable findings have also been reported in chickpea (Kumar and Abbo, 2001; Sundaram et al., 2019) and faba beans (Alghamdi, 2007). Likewise, the reproductive phases has been identified as a critical period for yield determination in chickpea, with grain number and yield being strongly influenced by both its duration and the environmental conditions prevailing this stage (Lake et al., 2014). However, previous studies have shown that early flowering does not necessarily translate into a longer reproductive duration or higher grain yield under all environmental conditions (Gaur et al., 2015a). Consistent with this observation, the weak environment-specific correlations detected in our study suggest that environmental conditions strongly influence the relationships among flowering time, seed-filling duration, and grain yield are strongly influenced by environmental conditions.

Plant architecture, particularly height, strongly affects productivity and harvest efficiency (Narnoliya et al., 2019). Taller and more erect plants improve light penetration and reduce canopy humidity, thereby limiting foliar diseases (Roorkiwal et al., 2020), which explains the strong positive correlation observed between GY and PH. Similar correlations have been reported in a previous study (Alemu et al., 2018). In our study, plant height and flowering earliness were the traits most strongly associated with seed yield, confirming their importance as key indicators of chickpea productivity. A significant reduction in PH and GY was recorded in Terbol, likely due to stressful climatic conditions. Similar stress-induced reductions in PH have been reported in chickpea (Rani et al., 2020) and lentil (Choukri et al., 2020). Furthermore, researchers have emphasized that unpredictable climate change poses a major constraint to chickpea production, increasing the frequency of droughts and extreme temperatures (<15 °C or >30 °C), significantly reducing seed yield (Kadiyala et al., 2016). High temperatures decrease both grain weight and number by reducing pollen viability and increasing flower and pod abortion in chickpeas (Rani et al., 2020). Water scarcity during the reproductive stages also reduces grain yield (El Sabagh et al., 2019) by severely affecting essential biological and biochemical processes. In these stressful conditions, it is important to select chickpea genotypes that are well adapted and capable of maintaining high, stable yields.

In Kabuli chickpea, the large seed size is considered one of the most important traits in the Mediterranean region. In our study, seed size (HSW), was positively correlated with D2F, while no correlation was observed between HSW and GY. This positive association between HSW and D2F suggests that selecting early flowering genotypes without compromising seed size may be challenging. A similar association has been reported by Saki et al. (2010). However, other studies have shown negative (Gaur et al., 2015b) or no correlation (Jivani et al., 2013) between D2F and HSW, indicating that combining early flowering with large seed size remains possible in certain genetic backgrounds.

### 4.2 Population structure and linkage disequilibrium

Identifying and selecting candidate genes and causal markers are key to developing successful germplasm. To effectively use genetic material in breeding programs, it is essential to understand genetic diversity, linkage disequilibrium, and population structure (Yu et al., 2006). In this study, 24,675 SNPs were used to characterize LD patterns and population structure in a chickpea MAGIC-subset population. In contrast to previous studies that reported clear genetic subdivision in chickpea, including three groups in Kujur et al. (2015b) and four in Farahani et al. (2019), our population did not display a strong genetic structure, although five clusters were detected by LEA and DAPC. This observation is consistent with the results of Thudi et al., who also reported no clear structuring (K = 13) in a chickpea MAGIC population (Thudi et al., 2023). The limited structure observed reflects the extensive genomic reshuffling typical of MAGIC populations. Multiple rounds of intercrossing among several founders generate a high number of recombination events per recombinant inbred line, as predicted by simulation studies (Ladejobi et al., 2016). This recombination-rich framework promotes strong allele mixing, reduces long-range LD, and improves fine-mapping resolution. LD decay analysis showed a high initial r² (~0.45) decreasing to 0.1 at 3.3 Mb, reflecting extended haplotype blocks typical of a self-pollinating species. However, LD decayed more slowly than in some other MAGIC populations (Thudi et al., 2023). This may be due to using only 165 RILs rather than the full set of 3053 lines, thereby capturing fewer recombination events. Despite this slower decay, the observed LD patterns remain adequate for GWAS and support the identification of genomic regions associated with agronomic traits.

### 4.3 Associations mapping

MAGIC populations provide high allelic diversity and recombination, improving the resolution and reliability of association studies and accelerating the identification and deployment of favorable alleles in breeding programs (Roorkiwal et al., 2020). To increase confidence in the detected associations, only MTAs that were consistently recovered across multiple analytical approaches were considered reliable, thereby ensuring stronger biological relevance and reducing the likelihood of false positives. A major association hotspot was detected on chromosome 4 (11.97–13.63 Mb), consistently associated with multiple traits across environments, while environment-specific MTAs revealed strong genotype × environment interactions. In our study, we identified 131 significant markers associated with phenological traits. Flowering time (D2F) was associated with 58 MTAs distributed mainly on chromosomes 1, 4, 6, 7, and 8, with only two detected in Terbol, indicating a marked environmental effect on flowering regulation. Similar findings have been reported in other chickpea MAGIC populations and GWAS studies (Srungarapu et al., 2022; Akinlade et al., 2025), confirming the polygenic and environment-dependent nature of this trait.

In total 44 MTAs (14 reliable) were identified for D2M on all chromosomes except 2, 3, and 8. These loci largely agree with published quantitative trait loci (QTLs) (Lichtenzveig et al., 2005; Pushpavalli et al., 2015; Srungarapu et al., 2022), although their slightly shifted positions suggest the presence of additional or novel genomic regions. In contrast to D2F and D2M, fewer studies have focused on F2M. Earlier works identified loci on chromosomes 3 and 4 (Lichtenzveig et al., 2005; Thudi et al., 2023)). In our analysis, 41 MTAs (13 reliable) were detected at Marchouch and Terbol, mainly on chromosomes 1, 2, 4, 6, and 7, which may also represent new *loci*. Several pleiotropic SNPs were identified in Marchouch, including loci shared between D2F and F2M on chromosome 1 (1.79–1.84 Mbp), and between D2M and F2M on chromosome 4 (14.66 Mb), as well as two loci common to D2M, F2M, and HSW (14.88–14.89 Mb). The co-localization of these genomic regions, particularly on chromosome 4, suggests the presence of major genes with pleiotropic effects (Chen and Lübberstedt, 2010) or clusters of tightly linked genes affecting phenological development. This region (spanning approximately 9.1–16.1 Mb) has repeatedly been reported as a genomic hotspot controlling flowering and yield-related traits (Varshney et al., 2014; Kale et al., 2015), a finding strongly corroborated by our results. For PH, 38 MTAs (30 reliable) were identified across environments, mainly on chromosomes 1, 4, 7, and 8. Fewer associations were detected at Terbol, consistent with reduced phenotypic variation under stressful conditions. As in previous studies (Narnoliya et al., 2019; Barmukh et al., 2021; Akinlade et al., 2025), PH exhibited a highly polygenic architecture.

Grain yield in chickpea is a highly complex trait resulting from the interaction of multiple agronomic and physiological components, making the identification of consistent loci across environments particularly challenging. In this study, 17 reliable MTAs were identified for GY at Marchouch, mainly on chromosomes 4 and 7, while only one association was detected at Terbol on chromosome 2 (35.92 Mbp). The low number of significant loci, particularly at Terbol, highlights the strong environmental dependency and low heritability of yield. Similar results have been reported in previous studies identifying QTLs for GY on chromosomes 1, 2, 4, 6, and 7 (Basu et al., 2019; Barmukh et al., 2021; Li et al., 2022), supporting our observations. The seed weight, that was a major determinant of grain yield, displayed extensive genetic control. At Marchouch, 213 MTAs (168 reliable) and at Terbol, 151 MTAs (82 reliable) were distributed across most chromosomes. A major and stable hotspot on chromosome 4 (11.97–13.63 Mb), comprising 57 reliable MTAs, overlapped with previously described regions governing seed weight (Thudi et al., 2016; Srungarapu et al., 2022). Several co-localized MTAs for PH and HSW suggest pleiotropic or tightly linked loci that simultaneously influence plant architecture and seed development. This hotspot represents a particularly promising target for marker-assisted selection aimed at improving both yield potential and seed quality.

Identification of candidate genes (CGs) is essential for understanding the molecular mechanisms underlying complex traits. In this study, several CGs associated with phenological, and agronomic traits encode proteins involved in kinase signaling, transcriptional regulation, stress response, and metabolic pathways. Many MTAs were located within genes showing pleiotropic effects, suggesting potential co-localization of genomic regions associated with multiple traits rather than demonstrating coordinated regulation of growth, development, and seed size.

Several MTAs associated with D2F, were found within receptor-like serine/threonine kinases on chromosome 1 (*Ca_00217*, *Ca_00218*, *Ca_00219*), which have been reported to regulate cell expansion and meristem activity (Brueggeman et al., 2002). Additional loci corresponded to genes with annotated functions related to related RNA processing (*Ca_00246*), quinone oxidoreductase (*Ca_00250*), amino acid transport (*Ca_00253*), and sulfur metabolism (*Ca_00254*). Candidate genes shared between D2F and F2M, such as *Ca_00226* and *Ca_00228*, encode cyclin D6–1 and nucleotide transporters, whose annotated functions suggest possible links to cell-cycle regulation and nucleotide flux in the floral transition (Inagaki and Umeda, 2011). For D2M, significant MTAs were located within genes with annotated functions related to nucleocytoplasmic transport (*Ca_13893*), chromatin organization (*Ca_06519* and *Ca_06527*), and phosphoinositide signaling (*Ca_17643*). These findings support their involvement in maturation processes that rely on active cell division and nuclear remodeling (Kumar and Kumar, 2024). For F2M, MTAs on chromosome 4 included genes encoding a serine/threonine kinase ATR and a mechanosensitive ion channel (*Ca_05391*, *Ca_05390*), which have been implicated in DNA damage signaling and mechanotransduction during reproductive development. For PH, MTAs on chromosomes 1 and 4 highlighted genes related to chromatin remodeling (*Ca_07088*), hormonal regulation and cell wall biosynthesis (*Ca_06968*, *Ca_06956*), and protein turnover/signaling (*Ca_04384*, *Ca_04492*), supporting their roles in stem elongation and architecture (Liu et al., 2016).

In addition, multiple CGs point to candidate biological processes potentially associated with seed size (HSW) and biomass accumulation. These include genes involved in redox regulation and defense (*Ca_04475*, *Ca_04424*) (Nabi et al., 2020), cytoskeletal organization (*Ca_07899*), oxidative stress tolerance (*Ca_07907*), and carbohydrate metabolism (*Ca_09577*). A genomic region on chromosome 4 (4:12004209–4:12589511) harbors several defense and growth-related genes, such as TIC32, C2H2 zinc finger proteins, and F-box/LRR proteins. These transcription factors likely coordinate developmental and stress-response pathways (Jiang et al., 2017). Several CGs, including *Ca_04424* and *Ca_04475*, showed pleiotropic associations with both HSW and PH, suggesting that this genomic region may harbor factors influencing multiple agronomic traits. Similarly, flowering and maturity-related genes such as *Ca_00226* and *Ca_00228* indicating possible shared genetic control among developmental and metabolic traits.

Overall, the identified CGs provide putative insights into the genetic architecture underlying key agronomic traits in chickpea. Based on their functional annotations, these genes may be related to pathways involving cell cycle regulation, transcriptional regulation, cytoskeleton organization, redox balance, and stress signaling. However, these inferences are based primarily based on positional proximity to significant SNPs and gene annotation information and therefore require further validation through complementary approaches, such as fine mapping, expression analyses, and functional studies.

## 5 Conclusion

Chickpea is an important legume crop for human nutrition. Superior chickpea lines with good agronomic performance and resilience to environmental stresses are in high demand by farmers. This study was conducted to assess the genetic variability of agronomic traits in a MAGIC-Subset population of chickpea grown in two Mediterranean environments: Marchouch, Morocco, and Terbol, Lebanon. The study revealed significant genetic variation among the tested genotypes with a significant genotype-environment interaction. Results showed earlier flowering under Marchouch conditions compared to the Terbol environment, associated with a significant increase in grain yield (66%). Superior genotypes (M-1407, M-2038, M-2079, M-242, M-2551, and M-987) with good agronomic performance (early flowering and high GY) were selected at both sites. These lines represents promising breeding materials with potential to become candidate varieties after further multi-environment and multi-year validation. GWAS analyses revealed both environment-specific and shared MTAs across sites. A total of 57 stable MTAs for HSW were identified across environments, mainly clustered within a major hotspot on chromosome 4 (11.97 - 13.63 Mbp), including pleiotropic loci controlling yield-related traits (HSW and PH). This concordance of MTA across both environments suggests cross-validation of their respective associations with the phenotypic traits studied, thereby reinforcing their reliability and relevance for further analyses. Annotation of associated markers identified candidate genes regulating several cellular functions and involved in growth regulation, signaling, and stress responses. The MTAs and candidate genes identified in this study provide an important resource to advance genetic research. However, such results can be supported by further validation using of the selected MTAs using different populations and diverse environments.

## Data Availability

Publicly available datasets were analyzed in this study. This data can be found here: https://doi.org/10.5281/zenodo.18656847.

## Footnotes

1
https://github.com/XiaoleiLiuBio/MVP

2
https://www.pulsedb.org/organism/641

3
http://www.uniprot.org
