# Supplementary material for: Multi-model genome-wide association analysis of agronomic traits in a Kabuli chickpea MAGIC-subset population (Cicer arietinum L.) across Mediterranean environments
Source: Front Plant Sci. 2026 Jul 21;17:1860578. doi: 10.3389/fpls.2026.1860578 (PMC13433441; doi:10.3389/fpls.2026.1860578)
Supplement: Supplementary file 1 [file SupplementaryFile1.docx]

**Supplementary Figure 1**. Schematic representation of the genotyping workflow. Overview of the GBS-t-based SNP discovery, target enrichment assay design, capture-based sequencing, SNP filtering, and LD pruning steps used prior to GWAS analyses.
