# Supplementary material for: Multi-model genome-wide association analysis of agronomic traits in a Kabuli chickpea MAGIC-subset population (Cicer arietinum L.) across Mediterranean environments
Source: Front Plant Sci. 2026 Jul 21;17:1860578. doi: 10.3389/fpls.2026.1860578 (PMC13433441; doi:10.3389/fpls.2026.1860578)
Supplement: Supplementary file 2 [file SupplementaryFile2.docx]

**Supplementary Figure 2.** Density distributions of the agronomic traits measured across chickpea genotypes grown at the Marchouch and Terbol sites. Number of days to flowering (D2F), number of days to maturity (D2M), number of days from flowering to maturity (F2M), plant height (PH), grain yield (GY), and hundred seed weight (HSW).
