# Supplementary material for: Multi-model genome-wide association analysis of agronomic traits in a Kabuli chickpea MAGIC-subset population (Cicer arietinum L.) across Mediterranean environments
Source: Front Plant Sci. 2026 Jul 21;17:1860578. doi: 10.3389/fpls.2026.1860578 (PMC13433441; doi:10.3389/fpls.2026.1860578)
Supplement: Supplementary file 3 [file SupplementaryFile3.docx]

**Supplementary Figure 3.** Population structure of the chickpea MAGIC-subset population.
(A) Bar plot of individual ancestry proportions for the genetic clusters inferred using the LEA package in R (K = 5). (B) Discriminant analysis of principal components clustering the population into five groups. (C) Unrooted phylogenetic tree depicting genetic relationships among the chickpea lines based on Nei’s genetic distance using 24,675 high-quality SNPs.
