# Supplementary material for: Multi-model genome-wide association analysis of agronomic traits in a Kabuli chickpea MAGIC-subset population (Cicer arietinum L.) across Mediterranean environments
Source: Front Plant Sci. 2026 Jul 21;17:1860578. doi: 10.3389/fpls.2026.1860578 (PMC13433441; doi:10.3389/fpls.2026.1860578)
Supplement: Supplementary file 6 [file SupplementaryFile6.docx]

**Supplementary Figure 6.** Manhattan and quantile-quantile plots depicting results of genome-wide association studies for yield related traits by GLM, MLM and FarmCPU (from center to outside) models using K and K + PCA statistical approaches at Marchouch.
