# Supplementary material for: Multi-model genome-wide association analysis of agronomic traits in a Kabuli chickpea MAGIC-subset population (Cicer arietinum L.) across Mediterranean environments
Source: Front Plant Sci. 2026 Jul 21;17:1860578. doi: 10.3389/fpls.2026.1860578 (PMC13433441; doi:10.3389/fpls.2026.1860578)
Supplement: Supplementary file 8 [file Table1.docx]

**Supplementary Table 1.** List of parental lines used to develop the MAGIC population.

| **Genotype** | **Species/ Botanical Group** | **Pedigree** | **Geographic Origin / Source Institution** | **Key Agronomic Traits** | **Resistance / tolerance to major biotic and abiotic stresses** |
| --- | --- | --- | --- | --- | --- |
| **ILC588** | *C. arietinum* L. (Kabuli) | - | India / ICRISAT | Large seed size, yield potential, Adaptation to Mediterranean environments | Ascochyta blight, Fusarium wilt, Drought |
| **ILC533** | *C. arietinum* L. (Kabuli) | - | Syria / ICARDA | Yield stability, Adaptation to mediterranean environments | Ascochyta blight, Drought |
| **ILWC81** | *Cicer reticulatum* L. | - | Turkey / ICARDA | Source for novel allelic diversity | Resistance to multiple diseases (Ascochyta blight, Botrytis gray mold), Drought, Cold tolerance |
| **ILWC292** | *Cicer reticulatum* L. | - | Turkey / ICARDA | Source for novel allelic diversity | Ascochyta blight, Root diseases, Drought |
| **FLIP91-96C** | *C. arietinum* L. (Kabuli) | X88TH19 / ICC 14218 × FLIP 84-93C | ICARDA breeding line | High yield, adaptation | Ascochyta blight, Heat, Drought |
| **X09TH77** | *C. arietinum* L. (Desi) | X09TH77 / FLIP91-96 × ICCV2 | Ethiopia/ICARDA | Yield potential under stress conditions | Disease tolerance, Drought |
| **ICC4958** | *C. arietinum* L. (Desi) | - | India / ICRISAT | Deep and prolific root system | Fusarium wilt, Drought |
| **Sel95TH1745** | *C. arietinum* L. (Desi) | - | Syria/ICARDA | Yield and adaptation | Ascochyta blight, Drought, Cold tolerance |
| **KAK-2** | *C. arietinum* L. (Desi) | - | India / ICRISAT | Early maturity, high yield | Fusarium wilt, Drought, |
| **FLIP03-98C** | *C. arietinum* L. (Kabuli) | X00TH49 / FLIP98-52C × FLIP98-10C | ICARDA breeding line | Yield potential, seed quality | Ascochyta blight, Fusarium wilt, Heat, Drought |
| **S080** | *C. arietinum* L. (Desi) | X00TH80 × 99TH31 × S99442 | Ethiopia / ICARDA | - | Disease tolerance |
| **FLIP03-121C** | *C. arietinum* L. (Kabuli) | X00TH51 / FLIP98-52C × FLIP98-47C | ICARDA breeding line | Yield stability, seed quality | Ascochyta blight, Fusarium wilt, drought, Heat |

Different crosses combinations: Cross1: ILC588xICC4958; Cross2: ILC533xSel 95TH1745; Cross3: ILWC81xKAK-2; Cross4: FLIP91-96xFLIP03-98C; Cross5: X09TH77xS080; Cross6: ILWC292xFLIP03-121C
