# Supplementary material for: Multi-model genome-wide association analysis of agronomic traits in a Kabuli chickpea MAGIC-subset population (Cicer arietinum L.) across Mediterranean environments
Source: Front Plant Sci. 2026 Jul 21;17:1860578. doi: 10.3389/fpls.2026.1860578 (PMC13433441; doi:10.3389/fpls.2026.1860578)
Supplement: Supplementary file 9 [file Table2.docx]

**Table S2.** Annotation of associated SNPs for agronomic traits

| **Associated traits** | **Environments** | **Models** | **SNP** | **Chromosomes** | **SNP physical positions (bp)** | **Gene ID** | **Gene Location** | **Sequence description** |
| --- | --- | --- | --- | --- | --- | --- | --- | --- |
| D2F | MCH | M1 M2 | 1:1732756 | 1 | 1732756 | Ca_00217 | Ca1:1731248..1734117 | Receptor-like serine/threonine-protein kinase SD1-8 |
| D2F | MCH | M1 M2 | 1:1736317 | 1 | 1736317 | _ | _ | _ |
| D2F | MCH | M1 M2 | 1:1742095 | 1 | 1742095 | Ca_00218 | Ca1:1738772..1742471 | Receptor-like serine/threonine-protein kinase SD1-8 |
| D2F | MCH | M1 M2 | 1:1746616 | 1 | 1746616 | Ca_00219 | Ca1:1742975..1749151 | Receptor-like serine/threonine-protein kinase SD1-8 |
| D2F | MCH | M1 M2 M3 M4 M5 | 1:1851565 | 1 | 1851565 | _ | _ | _ |
| D2F | MCH | M1 M2 M4 M5 | 1:1969497 | 1 | 1969497 | _ | _ | _ |
| D2F | MCH | M1 M2 M4 M5 | 1:1974676 | 1 | 1974676 | Ca_00246 | Ca1:1972074..1977002 | Pre-mRNA-splicing factor SYF1 |
| D2F | MCH | M1 M2 M4 M5 | 1:1979974 | 1 | 1979974 | _ | _ | _ |
| D2F | MCH | M1 M2 M4 M5 | 1:1982030 | 1 | 1982030 | _ | _ | _ |
| D2F | MCH | M1 M2 M4 M5 | 1:1983940 | 1 | 1983940 | _ | _ | _ |
| D2F | MCH | M1 M2 M4 M5 | 1:1985394 | 1 | 1985394 | _ | _ | _ |
| D2F | MCH | M1 M2 M4 M5 | 1:1992335 | 1 | 1992335 | _ | _ | _ |
| D2F | MCH | M1 M2 M4 M5 | 1:1996192 | 1 | 1996192 | Ca_00250 | Ca1:1995579..1998527 | Quinone oxidoreductase PIG3 |
| D2F | MCH | M1 M2 M4 M5 | 1:2000511 | 1 | 2000511 | _ | _ | _ |
| D2F | MCH | M1 M2 M4 M5 | 1:2006505 | 1 | 2006505 | _ | _ | _ |
| D2F | MCH | M1 M2 M4 M5 | 1:2007996 | 1 | 2007996 | _ | _ | _ |
| D2F | MCH | M1 M2 M4 M5 | 1:2013115 | 1 | 2013115 | Ca_00253 | Ca1:2011070..2015113 | Lysine histidine transporter-like 8 |
| D2F | MCH | M1 M2 M4 M5 | 1:2020243 | 1 | 2020243 | Ca_00254 | Ca1:2017338..2021198 | PAP-specific phosphatase HAL2-like protein |
| D2F | MCH | M1 M2 M4 M5 | 1:2023328 | 1 | 2023328 | _ | _ | _ |
| D2F | MCH | M1 M2 M4 M5 | 1:2025335 | 1 | 2025335 | _ | _ | _ |
| D2F | MCH | M1 M2 M4 M5 | 1:2030705 | 1 | 2030705 | _ | _ | _ |
| D2F | MCH | M1 M2 M4 M5 | 1:2031632 | 1 | 2031632 | _ | _ | _ |
| D2F | MCH | M1 M2 M4 M5 | 1:2032935 | 1 | 2032935 | _ | _ | _ |
| D2F | MCH | M1 M2 M4 M5 | 1:2044999 | 1 | 2044999 | _ | _ | _ |
| D2F | MCH | M1 M2 M4 M5 | 1:2054354 | 1 | 2054354 | _ | _ | _ |
| D2F | MCH | M1 M2 M4 M5 | 1:2065960 | 1 | 2065960 | _ | _ | _ |
| D2F | MCH | M1 M2 M4 M5 | 1:2077108 | 1 | 2077108 | _ | _ | _ |
| D2F | MCH | M1 M2 M4 M5 | 1:2083073 | 1 | 2083073 | Ca_00265 | Ca1:2079616..2083394 | Lysine ketoglutarate reductase trans-splicing-like protein |
| D2F | MCH | M1 M2 M4 M5 | 1:2090234 | 1 | 2090234 | _ | _ | _ |
| D2F | MCH | M1 M2 M4 M5 | 1:2095732 | 1 | 2095732 | Ca_00266 | Ca1:2092119..2098332 | Uncharacterized protein |
| D2F | MCH | M1 M2 M4 M5 | 1:2101384 | 1 | 2101384 | _ | _ | _ |
| D2F | MCH | M1 M2 M4 M5 | 1:2114028 | 1 | 2114028 | _ | _ | _ |
| D2F | MCH | M1 M2 M4 M5 | 1:2116377 | 1 | 2116377 | _ | _ | _ |
| D2F | MCH | M1 M2 M4 M5 | 1:2119069 | 1 | 2119069 | _ | _ | _ |
| D2F | MCH | M1 M2 M4 M5 | 1:2123129 | 1 | 2123129 | _ | _ | _ |
| D2F | MCH | M1 M2 M4 M5 | 1:2132273 | 1 | 2132273 | _ | _ | _ |
| D2F | MCH | M1 M2 M4 M5 | 1:2133834 | 1 | 2133834 | _ | _ | _ |
| D2F | MCH | M1 M2 M4 M5 | 1:2135788 | 1 | 2135788 | _ | _ | _ |
| D2F | MCH | M1 M2 M4 M5 | 1:2138945 | 1 | 2138945 | _ | _ | _ |
| D2F | MCH | M1 M2 M4 M5 | 1:2140595 | 1 | 2140595 | _ | _ | _ |
| D2F | MCH | M1 M2 | 4:35659857 | 4 | 35659857 | _ | _ | _ |
| D2F | MCH | M1 M2 | 6:23929907 | 6 | 23929907 | _ | _ | _ |
| D2F | MCH | M1 M2 | 6:23950468 | 6 | 23950468 | _ | _ | _ |
| D2F | MCH | M1 M2 M4 M5 | 7:32658628 | 7 | 32658628 | _ | _ | _ |
| D2F | MCH | M1 M2 M4 M5 | 7:32687765 | 7 | 32687765 | _ | _ | _ |
| D2F | MCH | M1 M2 M4 M5 M6 | 7:35539422 | 7 | 35539422 | Ca_17761 | Ca7:35538277..35544107 | Desiccation protectant protein Lea14 homolog |
| D2F | MCH | M1 M2 | 8:6281461 | 8 | 6281461 | _ | _ | _ |
| D2M, F2M | MCH | M1 M2 M4 M5 | 1:1790985 | 1 | 1790985 | _ | _ | _ |
| D2M, F2M | MCH | M1 M2 M4 M5 | 1:1791895 | 1 | 1791895 | Ca_00226 | Ca1:1791428..1793493 | Putative cyclin-D6-1 |
| D2M, F2M | MCH | M1 M2 M4 M5 | 1:1795804 | 1 | 1795804 | _ | _ | _ |
| D2M, F2M | MCH | M1 M2 M4 M5 | 1:1803125 | 1 | 1803125 | Ca_00228 | Ca1:1801332..1803667 | Equilibrative nucleotide transporter 3 |
| D2M, F2M | MCH | M1 M2 M4 M5 | 1:1806526 | 1 | 1806526 | _ | _ | _ |
| D2M, F2M | MCH | M1 M2 M4 M5 | 1:1808047 | 1 | 1808047 | _ | _ | _ |
| D2M, F2M | MCH | M1 M2 M4 M5 | 1:1836592 | 1 | 1836592 | _ | _ | _ |
| D2M, F2M | MCH | M1 M2 M4 M5 | 1:1844901 | 1 | 1844901 | _ | _ | _ |
| D2M, F2M | MCH | M1 M2 M4 M5 | 1:1848065 | 1 | 1848065 | _ | _ | _ |
| D2F | TBL | M1 M2 | 4:40879003 | 4 | 40879003 | _ | _ | _ |
| D2F | TBL | M1 M2 | 4:40880138 | 4 | 40880138 | _ | _ | _ |
| D2M | MCH | M1 M2 | 1:40015988 | 1 | 40015988 | _ | _ | _ |
| D2M | MCH | M1 M2 | 1:40045167 | 1 | 40045167 | _ | _ | _ |
| D2M | MCH | M1 M2 | 1:40056643 | 1 | 40056643 | _ | _ | _ |
| D2M | MCH | M1 M2 | 1:40070408 | 1 | 40070408 | _ | _ | _ |
| D2M | MCH | M1 M2 | 1:40084593 | 1 | 40084593 | _ | _ | _ |
| D2M | MCH | M1 M2 | 1:40099059 | 1 | 40099059 | _ | _ | _ |
| D2M | MCH | M1 M2 | 1:40113680 | 1 | 40113680 | _ | _ | _ |
| D2M | MCH | M1 M2 | 1:40140506 | 1 | 40140506 | _ | _ | _ |
| D2M | MCH | M1 M2 | 1:40159083 | 1 | 40159083 | _ | _ | _ |
| D2M | MCH | M1 M2 | 1:40182293 | 1 | 40182293 | _ | _ | _ |
| D2M | MCH | M1 M2 | 1:40339584 | 1 | 40339584 | Ca_13893 | Ca1:40335262..40369893 | Nuclear-pore anchor |
| D2M | MCH | M1 M2 | 1:40350639 | 1 | 40350639 | Ca_13893 | Ca1:40335262..40369893 | Nuclear-pore anchor |
| D2M | MCH | M1 M2 | 1:40359011 | 1 | 40359011 | Ca_13893 | Ca1:40335262..40369893 | Nuclear-pore anchor |
| D2M | MCH | M1 M2 | 1:40371578 | 1 | 40371578 | _ | _ | _ |
| D2M | MCH | M1 M2 M3 | 1:46385808 | 1 | 46385808 | _ | _ | _ |
| D2M | MCH | M1 M2 M3 | 4:12518917 | 4 | 12518917 | _ | _ | _ |
| D2M | MCH | M1 M2 | 4:28386756 | 4 | 28386756 | _ | _ | _ |
| D2M, F2M | MCH | M1 M2 M4 M5 | 4:14668376 | 4 | 14668376 | _ | _ | _ |
| D2M, F2M, HSW | MCH | M1 M2 M4 M5 M6 | 4:14884501 | 4 | 14884501 | _ | _ | _ |
| D2M, F2M, HSW | MCH | M1 M2 M4 M5 M6 | 4:14895497 | 4 | 14895497 | _ | _ | _ |
| D2M | TBL | M1 M2 M4 M5 M6 | 5:150469 | 5 | 150469 | _ | _ | _ |
| D2M | TBL | M1 M2 M4 M5 M6 | 5:213312 | 5 | 213312 | _ | _ | _ |
| D2M | TBL | M1 M2 M4 M5 M6 | 5:224696 | 5 | 224696 | _ | _ | _ |
| D2M | TBL | M1 M2 M3 M4 M5 M6 | 5:27417480 | 5 | 27417480 | _ | _ | _ |
| D2M | TBL | M1 M2 | 6:18800572 | 6 | 18800572 | _ | _ | _ |
| D2M | TBL | M1 M2 | 6:18877187 | 6 | 18877187 | _ | _ | _ |
| D2M | TBL | M1 M2 | 6:18886650 | 6 | 18886650 | _ | _ | _ |
| D2M | TBL | M1 M2 | 6:18958527 | 6 | 18958527 | _ | _ | _ |
| D2M | TBL | M1 M2 | 6:18966861 | 6 | 18966861 | _ | _ | _ |
| D2M | TBL | M1 M2 | 6:18969022 | 6 | 18969022 | _ | _ | _ |
| D2M | TBL | M1 M2 | 6:19021282 | 6 | 19021282 | _ | _ | _ |
| D2M | TBL | M1 M2 | 6:19066559 | 6 | 19066559 | _ | _ | _ |
| D2M | TBL | M1 M2 | 6:19097134 | 6 | 19097134 | _ | _ | _ |
| D2M | TBL | M1 M2 | 6:19128337 | 6 | 19128337 | Ca_06519 | Ca6:19127666..19139443 | Probable protein NAP1 |
| D2M | TBL | M1 M2 | 6:19147299 | 6 | 19147299 | _ | _ | _ |
| D2M | TBL | M1 M2 | 6:19190485 | 6 | 19190485 | Ca_06527 | Ca6:19187774..19191786 | Uncharacterized protein At4g19900 |
| D2M | TBL | M1 M2 | 6:19208970 | 6 | 19208970 | _ | _ | _ |
| D2M | TBL | M1 M2 | 6:7560540 | 6 | 7560540 | Ca_09627 | Ca6:7550395..7578261 | Spatacsin |
| D2M | TBL | M1 M2 | 6:7571776 | 6 | 7571776 | Ca_09627 | Ca6:7550395..7578261 | Spatacsin |
| D2M | TBL | M1 M2 M4 M5 M6 | 7:35226247 | 7 | 35226247 | _ | _ | _ |
| D2M | TBL | M1 M2 M4 M5 M6 | 7:35231894 | 7 | 35231894 | _ | _ | _ |
| D2M | TBL | M1 M2 M4 M5 M6 | 7:35239142 | 7 | 35239142 | Ca_17643 | Ca7:35238994..35241717 | Phosphatidylinositol 4-kinase gamma 4 |
| D2M | TBL | M1 M2 M4 M5 M6 | 7:35298751 | 7 | 35298751 | _ | _ | _ |
| D2M | TBL | M1 M2 M4 M5 M6 | 7:38357828 | 7 | 38357828 | _ | _ | _ |
| F2M | MCH | M1 M2 M3 M4 M5 M6 | 1:2768253 | 1 | 2768253 | _ | _ | _ |
| F2M | MCH | M1 M2 M3 | 4:17185071 | 4 | 17185071 | _ | _ | _ |
| F2M | MCH | M1 M2 | 4:17185444 | 4 | 17185444 | _ | _ | _ |
| F2M | MCH | M1 M2 | 4:17295946 | 4 | 17295946 | Ca_05392 | Ca4:17294283..17298743 | Putative uncharacterized protein |
| F2M | MCH | M1 M2 | 4:17305839 | 4 | 17305839 | Ca_05391 | Ca4:17299744..17313073 | Serine/threonine-protein kinase ATR |
| F2M | MCH | M1 M2 | 4:17317597 | 4 | 17317597 | Ca_05390 | Ca4:17316352..17320855 | Mechanosensitive ion channel protein 6 |
| F2M | MCH | M1 M2 | 4:17322236 | 4 | 17322236 | _ | _ | _ |
| F2M | MCH | M1 M2 | 4:17325551 | 4 | 17325551 | _ | _ | _ |
| F2M | MCH | M1 M2 | 4:17328562 | 4 | 17328562 | _ | _ | _ |
| F2M | MCH | M1 M2 | 4:17331132 | 4 | 17331132 | _ | _ | _ |
| F2M | MCH | M1 M2 | 4:17333781 | 4 | 17333781 | _ | _ | _ |
| F2M | MCH | M1 M2 | 4:17338753 | 4 | 17338753 | _ | _ | _ |
| F2M | MCH | M1 M2 | 4:17342177 | 4 | 17342177 | _ | _ | _ |
| F2M | MCH | M1 M2 | 4:17344319 | 4 | 17344319 | _ | _ | _ |
| F2M | MCH | M1 M2 | 4:17346408 | 4 | 17346408 | _ | _ | _ |
| F2M | MCH | M1 M2 | 4:17351372 | 4 | 17351372 | _ | _ | _ |
| F2M | MCH | M1 M2 | 4:17355029 | 4 | 17355029 | _ | _ | _ |
| F2M | MCH | M1 M2 | 4:17358098 | 4 | 17358098 | _ | _ | _ |
| F2M | MCH | M1 M2 | 4:17366584 | 4 | 17366584 | _ | _ | _ |
| F2M | MCH | M1 M2 | 4:18839683 | 4 | 18839683 | _ | _ | _ |
| F2M | MCH | M1 M2 M3 M4 M5 M6 | 4:20896684 | 4 | 20896684 | _ | _ | _ |
| F2M | MCH | M1 M2 M3 | 4:22784090 | 4 | 22784090 | _ | _ | _ |
| F2M | MCH | M1 M2 M4 M5 M6 | 7:35272202 | 7 | 35272202 | _ | _ | _ |
| F2M | TBL | M1 M2 M3 | 2:35392935 | 2 | 35392935 | _ | _ | _ |
| F2M | TBL | M1 M2 M3 | 2:35395080 | 2 | 35395080 | _ | _ | _ |
| F2M | TBL | M1 M2 M3 | 2:35921652 | 2 | 35921652 | _ | _ | _ |
| F2M | TBL | M1 M2 | 2:35921664 | 2 | 35921664 | _ | _ | _ |
| F2M | TBL | M1 M2 M3 M4 M5 M6 | 6:17255760 | 6 | 17255760 | _ | _ | _ |
| F2M | TBL | M1 M2 M3 | 6:17549812 | 6 | 17549812 | _ | _ | _ |
| PH | MCH | M1 M2 M4 M5 M6 | 1:14476967 | 1 | 14476967 | Ca_07088 | Ca1:14475350..14478200 | Histone deacetylase 8 |
| PH | MCH | M1 M2 M3 M4 M4 M6 | 1:16175217 | 1 | 16175217 | _ | _ | _ |
| PH | MCH | M1 M2 M3 M4 M4 M6 | 1:16201830 | 1 | 16201830 | _ | _ | _ |
| PH | MCH | M1 M2 M3 M4 M4 M6 | 1:16303178 | 1 | 16303178 | _ | _ | _ |
| PH | MCH | M1 M2 | 1:39498292 | 1 | 39498292 | _ | _ | _ |
| PH | MCH | M1 M2 | 1:39523339 | 1 | 39523339 | _ | _ | _ |
| PH | MCH | M1 M2 | 1:39589133 | 1 | 39589133 | _ | _ | _ |
| PH | MCH | M1 M2 | 1:39694089 | 1 | 39694089 | _ | _ | _ |
| PH | MCH | M1 M2 | 1:39836764 | 1 | 39836764 | _ | _ | _ |
| PH | MCH | M1 M2 | 1:39948097 | 1 | 39948097 | _ | _ | _ |
| PH | MCH | M1 M2 | 1:39981337 | 1 | 39981337 | _ | _ | _ |
| PH | MCH | M1 M2 | 1:40257496 | 1 | 40257496 | _ | _ | _ |
| PH | MCH | M1 M2 | 4:11479643 | 4 | 11479643 | _ | _ | _ |
| PH | MCH | M1 M2 | 4:11481102 | 4 | 11481102 | _ | _ | _ |
| PH | MCH | M1 M2 | 4:11490316 | 4 | 11490316 | Ca_04384 | Ca4:11489920..11491815 | Tubby-like F-box protein 8 |
| PH | MCH | M1 M2 | 4:11495309 | 4 | 11495309 | _ | _ | _ |
| PH | MCH | M1 M2 | 4:11504911 | 4 | 11504911 | _ | _ | _ |
| PH | MCH | M1 M2 | 4:11515443 | 4 | 11515443 | _ | _ | _ |
| PH | MCH | M1 M2 | 4:11519894 | 4 | 11519894 | _ | _ | _ |
| PH | MCH | M1 M2 | 4:11527769 | 4 | 11527769 | _ | _ | _ |
| PH | MCH | M1 M2 | 4:11529821 | 4 | 11529821 | _ | _ | _ |
| PH | MCH | M1 M2 | 4:11739851 | 4 | 11739851 | _ | _ | _ |
| PH | MCH | M1 M2 | 4:12691109 | 4 | 12691109 | _ | _ | _ |
| PH | MCH | M1 M2 | 4:12700518 | 4 | 12700518 | _ | _ | _ |
| PH | MCH | M1 M2 | 4:12713937 | 4 | 12713937 | _ | _ | _ |
| PH | MCH | M1 M2 M4 M5 | 4:12729275 | 4 | 12729275 | Ca_04492 | Ca4:12727172..12729286 | Inactive rhomboid protein 1 |
| PH | MCH | M1 M2 | 4:12745229 | 4 | 12745229 | _ | _ | _ |
| PH | MCH | M1 M2 M4 M5 | 7:18097288 | 7 | 18097288 | _ | _ | _ |
| PH | MCH | M1 M2 M4 M5 | 7:18323148 | 7 | 18323148 | _ | _ | _ |
| PH | MCH | M1 M2 M4 M5 | 7:18493051 | 7 | 18493051 | _ | _ | _ |
| PH | MCH | M1 M2 M4 M5 | 7:18691926 | 7 | 18691926 | _ | _ | _ |
| PH | MCH | M1 M2 M4 M5 | 7:18975256 | 7 | 18975256 | _ | _ | _ |
| PH | MCH | M1 M2 M4 M5 | 7:5825084 | 7 | 5825084 | _ | _ | _ |
| PH | MCH | M1 M2 M4 M5 | 7:5827859 | 7 | 5827859 | _ | _ | _ |
| PH | MCH | M1 M2 M4 M5 | 7:5872484 | 7 | 5872484 | _ | _ | _ |
| PH, HSW | MCH | M1 M2 M5 | 4:13668703 | 4 | 13668703 | _ | _ | _ |
| PH, HSW | MCH, TBL | M1 M2 | 1:38948224 | 1 | 38948224 | _ | _ | _ |
| PH, HSW | MCH, TBL | M1 M2 | 1:39004153 | 1 | 39004153 | _ | _ | _ |
| PH, HSW | MCH, TBL | M1 M2 | 1:39080953 | 1 | 39080953 | _ | _ | _ |
| PH, HSW | MCH, TBL | M1 M2 | 1:22154930 | 1 | 22154930 | _ | _ | _ |
| PH, HSW | MCH, TBL | M1 M2 M5 M6 | 4:11974769 | 4 | 11974769 | _ | _ | _ |
| PH, HSW | MCH, TBL | M1 M2 M5 M6 | 4:11993401 | 4 | 11993401 | _ | _ | _ |
| PH, HSW | MCH, TBL | M1 M2 M5 M6 | 4:12004209 | 4 | 12004209 | Ca_04424 | Ca4:12004042..12004857 | Protein SAR DEFICIENT 4 |
| PH, HSW | MCH, TBL | M1 M2 M5 M6 | 4:12021146 | 4 | 12021146 | _ | _ | _ |
| PH, HSW | MCH, TBL | M1 M2 M5 M6 | 4:12035077 | 4 | 12035077 | _ | _ | _ |
| PH, HSW | MCH, TBL | M1 M2 M5 M6 | 4:12043012 | 4 | 12043012 | _ | _ | _ |
| PH, HSW | MCH, TBL | M1 M2 M5 M6 | 4:12092336 | 4 | 12092336 | _ | _ | _ |
| PH, HSW | MCH, TBL | M1 M2 M5 M6 | 4:12107716 | 4 | 12107716 | _ | _ | _ |
| PH, HSW | MCH, TBL | M1 M2 M5 M6 | 4:12137077 | 4 | 12137077 | _ | _ | _ |
| PH, HSW | MCH, TBL | M1 M2 M5 M6 | 4:12156061 | 4 | 12156061 | _ | _ | _ |
| PH, HSW | MCH, TBL | M1 M2 M5 M6 | 4:12196961 | 4 | 12196961 | _ | _ | _ |
| PH, HSW | MCH, TBL | M1 M2 M5 M6 | 4:12207456 | 4 | 12207456 | _ | _ | _ |
| PH, HSW | MCH, TBL | M1 M2 M5 | 4:12261497 | 4 | 12261497 | Ca_04450 | Ca4:12261350..12266005 | Short-chain dehydrogenase TIC 32, chloroplastic |
| PH, HSW | MCH, TBL | M1 M2 M5 | 4:12270147 | 4 | 12270147 | Ca_04451 | Ca4:12267055..12278356 | Putative uncharacterized protein |
| PH, HSW | MCH, TBL | M1 M2 M5 | 4:12276180 | 4 | 12276180 | Ca_04451 | Ca4:12267055..12278356 | Putative uncharacterized protein |
| PH, HSW | MCH, TBL | M1 M2 M5 M6 | 4:12525792 | 4 | 12525792 | Ca_04475 | Ca4:12522142..12526684 | Transmembrane ascorbate ferrireductase 1 |
| PH, HSW | MCH, TBL | M1 M2 M5 M6 | 4:12529258 | 4 | 12529258 | _ | _ | _ |
| PH, HSW | MCH, TBL | M1 M2 M5 M6 | 4:12531983 | 4 | 12531983 | Ca_04476 | Ca4:12530698..12533201 | Remorin |
| PH, HSW | MCH, TBL | M1 M2 M5 M6 | 4:12536855 | 4 | 12536855 | _ | _ | _ |
| PH, HSW | MCH, TBL | M1 M2 M5 M6 | 4:12537807 | 4 | 12537807 | _ | _ | _ |
| PH, HSW | MCH, TBL | M1 M2 M5 M6 | 4:12539974 | 4 | 12539974 | _ | _ | _ |
| PH, HSW | MCH, TBL | M1 M2 M5 M6 | 4:12540752 | 4 | 12540752 | _ | _ | _ |
| PH, HSW | MCH, TBL | M1 M2 M5 M6 | 4:12541747 | 4 | 12541747 | _ | _ | _ |
| PH, HSW | MCH, TBL | M1 M2 M5 M6 | 4:12543527 | 4 | 12543527 | _ | _ | _ |
| PH, HSW | MCH, TBL | M1 M2 M5 M6 | 4:12544149 | 4 | 12544149 | _ | _ | _ |
| PH, HSW | MCH, TBL | M1 M2 M5 M6 | 4:12545777 | 4 | 12545777 | _ | _ | _ |
| PH, HSW | MCH, TBL | M1 M2 M5 M6 | 4:12549071 | 4 | 12549071 | _ | _ | _ |
| PH, HSW | MCH, TBL | M1 M2 M5 M6 | 4:12553504 | 4 | 12553504 | _ | _ | _ |
| PH, HSW | MCH, TBL | M1 M2 M5 M6 | 4:12558973 | 4 | 12558973 | Ca_04478 | Ca4:12556352..12560153 | Cleavage and polyadenylation specificity factor subunit 5 |
| PH, HSW | MCH, TBL | M1 M2 M5 M6 | 4:12561023 | 4 | 12561023 | _ | _ | _ |
| PH, HSW | MCH, TBL | M1 M2 M5 M6 | 4:12562268 | 4 | 12562268 | _ | _ | _ |
| PH, HSW | MCH, TBL | M1 M2 M5 M6 | 4:12563697 | 4 | 12563697 | _ | _ | _ |
| PH, HSW | MCH, TBL | M1 M2 M5 M6 | 4:12565417 | 4 | 12565417 | _ | _ | _ |
| PH, HSW | MCH, TBL | M1 M2 M5 M6 | 4:12568458 | 4 | 12568458 | Ca_04479 | Ca4:12568141..12569262 | Homoserine kinase |
| PH, HSW | MCH, TBL | M1 M2 M5 M6 | 4:12572117 | 4 | 12572117 | _ | _ | _ |
| PH, HSW | MCH, TBL | M1 M2 M5 M6 | 4:12573098 | 4 | 12573098 | _ | _ | _ |
| PH, HSW | MCH, TBL | M1 M2 M5 M6 | 4:12577739 | 4 | 12577739 | Ca_04480 | Ca4:12574148..12583660 | DNA mismatch repair protein MSH3 |
| PH, HSW | MCH, TBL | M1 M2 M5 M6 | 4:12582192 | 4 | 12582192 | Ca_04480 | Ca4:12574148..12583660 | DNA mismatch repair protein MSH3 |
| PH, HSW | MCH, TBL | M1 M2 M5 M6 | 4:12585287 | 4 | 12585287 | _ | _ | _ |
| PH, HSW | MCH, TBL | M1 M2 M5 M6 | 4:12586115 | 4 | 12586115 | _ | _ | _ |
| PH, HSW | MCH, TBL | M1 M2 M5 M6 | 4:12589511 | 4 | 12589511 | Ca_04481 | Ca4:12589488..12591048 | F-box/LRR-repeat protein At3g48880 |
| PH, HSW | MCH, TBL | M1 M2 M5 M6 | 4:12593611 | 4 | 12593611 | _ | _ | _ |
| PH, HSW | MCH, TBL | M1 M2 M5 M6 | 4:12596770 | 4 | 12596770 | _ | _ | _ |
| PH, HSW | MCH, TBL | M1 M2 M5 M6 | 4:12601057 | 4 | 12601057 | _ | _ | _ |
| PH, HSW | MCH, TBL | M1 M2 M5 M6 | 4:12604952 | 4 | 12604952 | _ | _ | _ |
| PH, HSW | MCH, TBL | M1 M2 M5 M6 | 4:12608866 | 4 | 12608866 | _ | _ | _ |
| PH, HSW | MCH, TBL | M1 M2 M5 M6 | 4:12622076 | 4 | 12622076 | _ | _ | _ |
| PH, HSW | MCH, TBL | M1 M2 M5 M6 | 4:12652217 | 4 | 12652217 | _ | _ | _ |
| PH, HSW | MCH, TBL | M1 M2 M5 M6 | 4:12665710 | 4 | 12665710 | _ | _ | _ |
| PH, HSW | MCH, TBL | M1 M2 M5 M6 | 4:12670663 | 4 | 12670663 | _ | _ | _ |
| PH, HSW | MCH, TBL | M1 M2 M5 | 4:12676230 | 4 | 12676230 | _ | _ | _ |
| PH, HSW | MCH, TBL | M1 M2 M5 | 4:12688331 | 4 | 12688331 | _ | _ | _ |
| PH, HSW | MCH, TBL | M1 M2 M5 | 4:13618449 | 4 | 13618449 | _ | _ | _ |
| PH, HSW | MCH, TBL | M1 M2 M5 | 4:13631940 | 4 | 13631940 | _ | _ | _ |
| PH | TBL | M1 M2 | 1:15790483 | 1 | 15790483 | Ca_06968 | Ca1:15787871..15788152 | COBRA-like protein 10 |
| PH | TBL | M1 M2 | 1:15947130 | 1 | 15947130 | _ | _ | _ |
| PH | TBL | M1 M2 | 1:15964219 | 1 | 15964219 | Ca_06956 | Ca1:15963878..15966101 | Transcription factor ILR3 |
| PH | TBL | M1 M2 | 1:15968344 | 1 | 15968344 | _ | _ | _ |
| PH | TBL | M1 M2 | 1:18319481 | 1 | 18319481 | _ | _ | _ |
| PH | TBL | M1 M2 | 8:10009121 | 8 | 10009121 | _ | _ | _ |
| PH | TBL | M1 M2 | 8:10071996 | 8 | 10071996 | _ | _ | _ |
| PH | TBL | M1 M2 | 8:10699600 | 8 | 10699600 | _ | _ | _ |
| GY | MCH | M1 M2 M3 | 4:37868277 | 4 | 37868277 | _ | _ | _ |
| GY | MCH | M1 M2 M3 | 4:38048225 | 4 | 38048225 | _ | _ | _ |
| GY | MCH | M1 M2 M3 M4 M5 M6 | 7:32840883 | 7 | 32840883 | _ | _ | _ |
| GY | MCH | M1 M2 M3 M4 M5 M6 | 7:32843156 | 7 | 32843156 | _ | _ | _ |
| GY | MCH | M1 M2 M3 M4 M5 M6 | 7:32847054 | 7 | 32847054 | _ | _ | _ |
| GY | MCH | M1 M2 M3 M4 M5 M6 | 7:32848797 | 7 | 32848797 | _ | _ | _ |
| GY | MCH | M1 M2 M3 M4 M5 M6 | 7:32850471 | 7 | 32850471 | _ | _ | _ |
| GY | MCH | M1 M2 M3 M4 M5 M6 | 7:32852700 | 7 | 32852700 | _ | _ | _ |
| GY | MCH | M1 M2 M3 M4 M5 M6 | 7:32857774 | 7 | 32857774 | _ | _ | _ |
| GY | MCH | M1 M2 M3 M4 M5 M6 | 7:32860869 | 7 | 32860869 | _ | _ | _ |
| GY | MCH | M1 M2 M3 M4 M5 M6 | 7:32868942 | 7 | 32868942 | _ | _ | _ |
| GY | MCH | M1 M2 M3 M4 M5 M6 | 7:32874436 | 7 | 32874436 | _ | _ | _ |
| GY | MCH | M1 M2 M3 M4 M5 M6 | 7:32878663 | 7 | 32878663 | _ | _ | _ |
| GY | MCH | M1 M2 M3 M4 M5 M6 | 7:32881056 | 7 | 32881056 | _ | _ | _ |
| GY | MCH | M1 M2 M3 M4 M5 M6 | 7:32881565 | 7 | 32881565 | _ | _ | _ |
| GY | MCH | M1 M2 M3 M4 M5 M6 | 7:32882769 | 7 | 32882769 | _ | _ | _ |
| GY | MCH | M1 M2 M3 M4 M5 M6 | 7:32883200 | 7 | 32883200 | _ | _ | _ |
| GY | TBL | M1 M2 M4 M5 M6 | 2:35921714 | 2 | 35921714 | _ | _ | _ |
| HSW | MCH | M1 M2 M5 | 1:22086811 | 1 | 22086811 | _ | _ | _ |
| HSW | MCH | M1 M2 M5 | 1:22504875 | 1 | 22504875 | _ | _ | _ |
| HSW | MCH | M1 M2 M3 M5 M6 | 1:39855206 | 1 | 39855206 | _ | _ | _ |
| HSW | MCH | M1 M2 M3 M5 M6 | 1:42166927 | 1 | 42166927 | _ | _ | _ |
| HSW | MCH | M1 M2 M3 M5 M6 | 1:42490809 | 1 | 42490809 | _ | _ | _ |
| HSW | MCH | M1 M2 M3 M5 M6 | 1:43243014 | 1 | 43243014 | _ | _ | _ |
| HSW | MCH | M1 M2 M3 M5 M6 | 1:6087970 | 1 | 6087970 | _ | _ | _ |
| HSW | MCH | M1 M2 M3 M5 M6 | 1:6090430 | 1 | 6090430 | _ | _ | _ |
| HSW | MCH | M1 M2 M3 M5 M6 | 1:6095361 | 1 | 6095361 | _ | _ | _ |
| HSW | MCH | M1 M2 M3 M5 M6 | 1:6310346 | 1 | 6310346 | _ | _ | _ |
| HSW | MCH | M1 M2 M3 M5 M6 | 1:6312803 | 1 | 6312803 | _ | _ | _ |
| HSW | MCH | M1 M2 M3 M5 M6 | 1:6317378 | 1 | 6317378 | Ca_07892 | Ca1:6314978..6320152 | Transcriptional adapter ADA2 |
| HSW | MCH | M1 M2 M3 M5 M6 | 1:6322137 | 1 | 6322137 | _ | _ | _ |
| HSW | MCH | M1 M2 M3 M5 M6 | 1:6325154 | 1 | 6325154 | _ | _ | _ |
| HSW | MCH | M1 M2 M3 M5 M6 | 1:6326314 | 1 | 6326314 | _ | _ | _ |
| HSW | MCH | M1 M2 M3 M5 M6 | 1:6329847 | 1 | 6329847 | Ca_07893 | Ca1:6328983..6333996 | Putative uncharacterized protein |
| HSW | MCH | M1 M2 M3 M5 M6 | 1:6334878 | 1 | 6334878 | _ | _ | _ |
| HSW | MCH | M1 M2 M3 M5 M6 | 1:6336546 | 1 | 6336546 | _ | _ | _ |
| HSW | MCH | M1 M2 M3 M5 M6 | 1:6338204 | 1 | 6338204 | _ | _ | _ |
| HSW | MCH | M1 M2 M3 M5 M6 | 1:6342151 | 1 | 6342151 | _ | _ | _ |
| HSW | MCH | M1 M2 M3 M5 M6 | 1:6344056 | 1 | 6344056 | _ | _ | _ |
| HSW | MCH | M1 M2 M3 M5 M6 | 1:6344745 | 1 | 6344745 | _ | _ | _ |
| HSW | MCH | M1 M2 M3 M5 M6 | 1:6347279 | 1 | 6347279 | _ | _ | _ |
| HSW | MCH | M1 M2 M3 M5 M6 | 1:6352037 | 1 | 6352037 | _ | _ | _ |
| HSW | MCH | M1 M2 M3 M5 M6 | 1:6354804 | 1 | 6354804 | _ | _ | _ |
| HSW | MCH | M1 M2 M3 M5 M6 | 1:6356930 | 1 | 6356930 | _ | _ | _ |
| HSW | MCH | M1 M2 M3 M5 M6 | 1:6358187 | 1 | 6358187 | _ | _ | _ |
| HSW | MCH | M1 M2 M3 M5 M6 | 1:6360357 | 1 | 6360357 | _ | _ | _ |
| HSW | MCH | M1 M2 M3 M5 M6 | 1:6361545 | 1 | 6361545 | _ | _ | _ |
| HSW | MCH | M1 M2 M3 M5 M6 | 1:6363016 | 1 | 6363016 | _ | _ | _ |
| HSW | MCH | M1 M2 M3 M5 M6 | 1:6364096 | 1 | 6364096 | _ | _ | _ |
| HSW | MCH | M1 M2 M3 M5 M6 | 1:6365233 | 1 | 6365233 | _ | _ | _ |
| HSW | MCH | M1 M2 M3 M5 M6 | 1:6366163 | 1 | 6366163 | _ | _ | _ |
| HSW | MCH | M1 M2 M3 M5 M6 | 1:6367084 | 1 | 6367084 | _ | _ | _ |
| HSW | MCH | M1 M2 M3 M5 M6 | 1:6367976 | 1 | 6367976 | _ | _ | _ |
| HSW | MCH | M1 M2 M3 M5 M6 | 1:6368977 | 1 | 6368977 | _ | _ | _ |
| HSW | MCH | M1 M2 M3 M5 M6 | 1:6370671 | 1 | 6370671 | _ | _ | _ |
| HSW | MCH | M1 M2 M3 M5 M6 | 1:6372889 | 1 | 6372889 | _ | _ | _ |
| HSW | MCH | M1 M2 M3 M5 M6 | 1:6374895 | 1 | 6374895 | _ | _ | _ |
| HSW | MCH | M1 M2 M3 M5 M6 | 1:6379850 | 1 | 6379850 | Ca_07899 | Ca1:6377341..6381420 | Microtubule-associated protein 70-5 |
| HSW | MCH | M1 M2 M3 M5 M6 | 1:6384832 | 1 | 6384832 | _ | _ | _ |
| HSW | MCH | M1 M2 M3 M5 M6 | 1:6388946 | 1 | 6388946 | _ | _ | _ |
| HSW | MCH | M1 M2 M3 M5 M6 | 1:6395207 | 1 | 6395207 | _ | _ | _ |
| HSW | MCH | M1 M2 M3 M5 M6 | 1:6398405 | 1 | 6398405 | _ | _ | _ |
| HSW | MCH | M1 M2 M3 M5 M6 | 1:6400572 | 1 | 6400572 | _ | _ | _ |
| HSW | MCH | M1 M2 M5 | 1:6416556 | 1 | 6416556 | Ca_07905 | Ca1:6413897..6417797 | Heavy metal-associated isoprenylated plant protein 8 |
| HSW | MCH | M1 M2 M5 | 1:6419995 | 1 | 6419995 | _ | _ | _ |
| HSW | MCH | M1 M2 M5 | 1:6421345 | 1 | 6421345 | _ | _ | _ |
| HSW | MCH | M1 M2 M5 | 1:6422624 | 1 | 6422624 | _ | _ | _ |
| HSW | MCH | M1 M2 M5 | 1:6424208 | 1 | 6424208 | _ | _ | _ |
| HSW | MCH | M1 M2 M3 M4 M5 M6 | 1:6425224 | 1 | 6425224 | _ | _ | _ |
| HSW | MCH | M1 M2 M3 M5 M6 | 1:6426087 | 1 | 6426087 | _ | _ | _ |
| HSW | MCH | M1 M2 M5 | 1:6426614 | 1 | 6426614 | _ | _ | _ |
| HSW | MCH | M1 M2 M3 M5 M6 | 1:6428561 | 1 | 6428561 | _ | _ | _ |
| HSW | MCH | M1 M2 M5 | 1:6428985 | 1 | 6428985 | _ | _ | _ |
| HSW | MCH | M1 M2 M3 M5 M6 | 1:6431450 | 1 | 6431450 | _ | _ | _ |
| HSW | MCH | M1 M2 M5 | 1:6431713 | 1 | 6431713 | _ | _ | _ |
| HSW | MCH | M1 M2 M3 M5 M6 | 1:6435331 | 1 | 6435331 | Ca_07907 | Ca1:6433769..6437379 | Nudix hydrolase 2 |
| HSW | MCH | M1 M2 M5 | 1:6436679 | 1 | 6436679 | Ca_07907 | Ca1:6433769..6437379 | Nudix hydrolase 2 |
| HSW | MCH | M1 M2 M3 M5 M6 | 1:6438774 | 1 | 6438774 | _ | _ | _ |
| HSW | MCH | M1 M2 M5 | 1:6439527 | 1 | 6439527 | _ | _ | _ |
| HSW | MCH | M1 M2 M3 M5 M6 | 1:6441884 | 1 | 6441884 | Ca_07908 | Ca1:6440030..6443204 | Trihelix transcription factor GT-2 |
| HSW | MCH | M1 M2 M3 M5 M6 | 1:6445007 | 1 | 6445007 | _ | _ | _ |
| HSW | MCH | M1 M2 M5 | 1:6445754 | 1 | 6445754 | _ | _ | _ |
| HSW | MCH | M1 M2 M3 M5 M6 | 1:6446963 | 1 | 6446963 | _ | _ | _ |
| HSW | MCH | M1 M2 M3 M5 M6 | 1:6452612 | 1 | 6452612 | _ | _ | _ |
| HSW | MCH | M1 M2 M5 | 1:6455480 | 1 | 6455480 | _ | _ | _ |
| HSW | MCH | M1 M2 M3 M5 M6 | 1:6455873 | 1 | 6455873 | _ | _ | _ |
| HSW | MCH | M1 M2 M3 M5 M6 | 1:6457469 | 1 | 6457469 | _ | _ | _ |
| HSW | MCH | M1 M2 M3 M5 M6 | 1:6460741 | 1 | 6460741 | _ | _ | _ |
| HSW | MCH | M1 M2 M3 M5 M6 | 1:6464407 | 1 | 6464407 | _ | _ | _ |
| HSW | MCH | M1 M2 M3 M5 M6 | 1:6464956 | 1 | 6464956 | _ | _ | _ |
| HSW | MCH | M1 M2 M3 M5 M6 | 1:6487945 | 1 | 6487945 | Ca_07911 | Ca1:6479886..6492951 | Sister chromatid cohesion protein PDS5 homolog B-B |
| HSW | MCH | M1 M2 M3 M5 M6 | 1:6489581 | 1 | 6489581 | Ca_07911 | Ca1:6479886..6492951 | Sister chromatid cohesion protein PDS5 homolog B-B |
| HSW | MCH | M1 M2 M3 M5 M6 | 1:6494903 | 1 | 6494903 | _ | _ | _ |
| HSW | MCH | M1 M2 M3 M5 M6 | 1:6496734 | 1 | 6496734 | _ | _ | _ |
| HSW | MCH | M1 M2 M3 M5 M6 | 1:6498820 | 1 | 6498820 | _ | _ | _ |
| HSW | MCH | M1 M2 M3 M5 M6 | 1:6498934 | 1 | 6498934 | _ | _ | _ |
| HSW | MCH | M1 M2 M3 M5 M6 | 1:6501111 | 1 | 6501111 | _ | _ | _ |
| HSW | MCH | M1 M2 M5 M6 | 1:6501153 | 1 | 6501153 | _ | _ | _ |
| HSW | MCH | M1 M2 M3 M5 M6 | 1:6501167 | 1 | 6501167 | _ | _ | _ |
| HSW | MCH | M1 M2 M3 M5 M6 | 1:6502730 | 1 | 6502730 | _ | _ | _ |
| HSW | MCH | M1 M2 M3 M5 M6 | 1:6504357 | 1 | 6504357 | _ | _ | _ |
| HSW | MCH | M1 M2 M3 M5 M6 | 1:6506219 | 1 | 6506219 | _ | _ | _ |
| HSW | MCH | M1 M2 M3 M5 M6 | 1:6506968 | 1 | 6506968 | _ | _ | _ |
| HSW | MCH | M1 M2 M3 M5 M6 | 1:6513217 | 1 | 6513217 | _ | _ | _ |
| HSW | MCH | M1 M2 M3 M5 M6 | 1:6514408 | 1 | 6514408 | _ | _ | _ |
| HSW | MCH | M1 M2 M3 M5 M6 | 1:6515689 | 1 | 6515689 | Ca_07915 | Ca1:6515188..6516879 | Alpha-ketoglutarate-dependent dioxygenase alkB homolog 2 |
| HSW | MCH | M1 M2 M3 M5 M6 | 1:6519787 | 1 | 6519787 | _ | _ | _ |
| HSW | MCH | M1 M2 M3 M5 M6 | 1:6528805 | 1 | 6528805 | _ | _ | _ |
| HSW | MCH | M1 M2 M3 M5 M6 | 1:6531173 | 1 | 6531173 | _ | _ | _ |
| HSW | MCH | M1 M2 M3 M5 M6 | 1:6533646 | 1 | 6533646 | _ | _ | _ |
| HSW | MCH | M1 M2 M5 M6 | 1:6535277 | 1 | 6535277 | _ | _ | _ |
| HSW | MCH | M1 M2 M3 M5 M6 | 1:6697867 | 1 | 6697867 | _ | _ | _ |
| HSW | MCH | M1 M2 M3 M5 M6 | 1:6699714 | 1 | 6699714 | _ | _ | _ |
| HSW | MCH | M1 M2 M3 M5 M6 | 1:6702841 | 1 | 6702841 | _ | _ | _ |
| HSW | MCH | M1 M2 M3 M5 M6 | 1:6708673 | 1 | 6708673 | _ | _ | _ |
| HSW | MCH | M1 M2 M3 M5 M6 | 1:6713011 | 1 | 6713011 | _ | _ | _ |
| HSW | MCH | M1 M2 M3 M5 M6 | 1:6714982 | 1 | 6714982 | _ | _ | _ |
| HSW | MCH | M1 M2 M3 M5 M6 | 1:6717879 | 1 | 6717879 | _ | _ | _ |
| HSW | MCH | M1 M2 M3 M5 M6 | 1:6737905 | 1 | 6737905 | _ | _ | _ |
| HSW | MCH | M1 M2 M3 M5 M6 | 1:6743428 | 1 | 6743428 | _ | _ | _ |
| HSW | MCH | M1 M2 M3 M5 M6 | 1:6747071 | 1 | 6747071 | _ | _ | _ |
| HSW | MCH | M1 M2 M3 M5 M6 | 1:6749627 | 1 | 6749627 | _ | _ | _ |
| HSW | MCH | M1 M2 M3 M5 M6 | 1:6760202 | 1 | 6760202 | _ | _ | _ |
| HSW | MCH | M1 M2 M3 M5 M6 | 1:6766113 | 1 | 6766113 | Ca_07941 | Ca1:6763496..6766527 | Uncharacterized protein At3g52155, chloroplastic |
| HSW | MCH | M1 M2 M3 M5 M6 | 1:6768145 | 1 | 6768145 | _ | _ | _ |
| HSW | MCH | M1 M2 M3 M5 M6 | 3:39318362 | 3 | 39318362 | _ | _ | _ |
| HSW | MCH | M1 M2 | 3:6588239 | 3 | 6588239 | _ | _ | _ |
| HSW | MCH | M1 M2 | 3:7657372 | 3 | 7657372 | _ | _ | _ |
| HSW | MCH | M1 M2 | 4:15761321 | 4 | 15761321 | _ | _ | _ |
| HSW | MCH | M1 M2 M3 M4 M5 M6 | 6:7953007 | 6 | 7953007 | _ | _ | _ |
| HSW | MCH | M1 M2 M5 | 6:7955434 | 6 | 7955434 | _ | _ | _ |
| HSW | MCH | M1 M2 | 6:7971285 | 6 | 7971285 | _ | _ | _ |
| HSW | MCH | M1 M2 | 6:7975311 | 6 | 7975311 | _ | _ | _ |
| HSW | MCH | M1 M2 | 6:7981537 | 6 | 7981537 | _ | _ | _ |
| HSW | MCH | M1 M2 | 6:7985194 | 6 | 7985194 | Ca_09577 | Ca6:7983136..7985505 | Probable trehalose-phosphate phosphatase I |
| HSW | MCH | M1 M2 | 6:7991073 | 6 | 7991073 | _ | _ | _ |
| HSW | MCH | M1 M2 | 6:7993515 | 6 | 7993515 | _ | _ | _ |
| HSW | MCH | M1 M2 | 6:7996596 | 6 | 7996596 | _ | _ | _ |
| HSW | MCH | M1 M2 | 6:7999834 | 6 | 7999834 | _ | _ | _ |
| HSW | MCH | M1 M2 | 6:8002709 | 6 | 8002709 | Ca_09574 | Ca6:8001481..8005207 | Protein SCO1 homolog 2, mitochondrial |
| HSW | MCH | M1 M2 | 6:8006443 | 6 | 8006443 | _ | _ | _ |
| HSW | MCH | M1 M2 | 6:8012131 | 6 | 8012131 | _ | _ | _ |
| HSW | MCH | M1 M2 | 6:8023976 | 6 | 8023976 | _ | _ | _ |
| HSW | MCH | M1 M2 | 6:8030210 | 6 | 8030210 | _ | _ | _ |
| HSW | MCH | M1 M2 | 6:8033936 | 6 | 8033936 | _ | _ | _ |
| HSW | MCH | M1 M2 | 6:8039633 | 6 | 8039633 | _ | _ | _ |
| HSW | MCH | M1 M2 | 6:8045078 | 6 | 8045078 | _ | _ | _ |
| HSW | MCH | M1 M2 | 6:8047827 | 6 | 8047827 | _ | _ | _ |
| HSW | MCH | M1 M2 | 6:8053188 | 6 | 8053188 | Ca_09568 | Ca6:8050243..8053706 | TLD domain-containing protein 2 |
| HSW | MCH | M1 M2 | 6:8061834 | 6 | 8061834 | _ | _ | _ |
| HSW | MCH | M1 M2 | 6:8065617 | 6 | 8065617 | _ | _ | _ |
| HSW | MCH | M1 M2 | 6:8076021 | 6 | 8076021 | Ca_09564 | Ca6:8075620..8078299 | Ras-related protein RABH1e |
| HSW | MCH | M1 M2 | 6:8079740 | 6 | 8079740 | _ | _ | _ |
| HSW | MCH | M1 M2 | 6:8086101 | 6 | 8086101 | _ | _ | _ |
| HSW | MCH | M1 M2 | 6:8088814 | 6 | 8088814 | Ca_09562 | Ca6:8087231..8093131 | Calmodulin-binding transcription activator 3 |
| HSW | MCH | M1 M2 | 6:8099471 | 6 | 8099471 | _ | _ | _ |
| HSW | MCH | M1 M2 | 6:8107699 | 6 | 8107699 | _ | _ | _ |
| HSW | MCH | M1 M2 | 6:8120270 | 6 | 8120270 | _ | _ | _ |
| HSW | MCH | M1 M2 | 6:8125843 | 6 | 8125843 | _ | _ | _ |
| HSW | MCH | M1 M2 | 6:8128062 | 6 | 8128062 | _ | _ | _ |
| HSW | MCH | M1 M2 | 6:8129183 | 6 | 8129183 | _ | _ | _ |
| HSW | MCH | M1 M2 | 6:8134567 | 6 | 8134567 | _ | _ | _ |
| HSW | MCH | M1 M2 | 6:8142809 | 6 | 8142809 | _ | _ | _ |
| HSW | MCH | M1 M2 | 6:8150697 | 6 | 8150697 | _ | _ | _ |
| HSW | MCH | M1 M2 | 6:8153672 | 6 | 8153672 | _ | _ | _ |
| HSW | MCH | M1 M2 | 6:8159937 | 6 | 8159937 | Ca_09554 | Ca6:8158931..8161229 | Monoacylglycerol lipase ABHD6 |
| HSW | MCH | M1 M2 | 6:8169198 | 6 | 8169198 | Ca_09552 | Ca6:8165843..8173016 | Enhancer of polycomb homolog 2 |
| HSW | MCH | M1 M2 | 6:8175813 | 6 | 8175813 | _ | _ | _ |
| HSW | MCH | M1 M2 | 6:8182306 | 6 | 8182306 | _ | _ | _ |
| HSW | MCH | M1 M2 M3 M6 | 7:15379725 | 7 | 15379725 | _ | _ | _ |
| HSW | MCH | M1 M2 M5 | 7:5411975 | 7 | 5411975 | _ | _ | _ |
| HSW | MCH, TBL | M1 M2 | 6:2529960 | 6 | 2529960 | _ | _ | _ |
| HSW | MCH, TBL | M1 M2 M5 | 6:2530350 | 6 | 2530350 | _ | _ | _ |
| HSW | TBL | M1 M2 | 1:19711803 | 1 | 19711803 | _ | _ | _ |
| HSW | TBL | M1 M2 | 1:19853669 | 1 | 19853669 | _ | _ | _ |
| HSW | TBL | M1 M2 | 1:19918701 | 1 | 19918701 | _ | _ | _ |
| HSW | TBL | M1 M2 | 1:22855438 | 1 | 22855438 | _ | _ | _ |
| HSW | TBL | M1 M2 | 1:23150169 | 1 | 23150169 | _ | _ | _ |
| HSW | TBL | M1 M2 | 1:23451631 | 1 | 23451631 | _ | _ | _ |
| HSW | TBL | M1 M2 | 1:23674534 | 1 | 23674534 | _ | _ | _ |
| HSW | TBL | M1 M2 | 1:23877820 | 1 | 23877820 | _ | _ | _ |
| HSW | TBL | M1 M2 | 1:24197323 | 1 | 24197323 | _ | _ | _ |
| HSW | TBL | M1 M2 | 1:25312617 | 1 | 25312617 | _ | _ | _ |
| HSW | TBL | M1 M2 | 1:25533631 | 1 | 25533631 | _ | _ | _ |
| HSW | TBL | M1 M2 | 1:25737022 | 1 | 25737022 | Ca_18581 | Ca1:25735679..25738799 | [Pyruvate dehydrogenase (acetyl-transferring)] kinase, mitochondrial |
| HSW | TBL | M1 M2 | 1:25999324 | 1 | 25999324 | _ | _ | _ |
| HSW | TBL | M1 M2 | 1:26155179 | 1 | 26155179 | Ca_18597 | Ca1:26149649..26156387 | Subtilisin-like protease SDD1 |
| HSW | TBL | M1 M2 | 1:27343960 | 1 | 27343960 | _ | _ | _ |
| HSW | TBL | M1 M2 | 1:27548388 | 1 | 27548388 | _ | _ | _ |
| HSW | TBL | M1 M2 | 1:27700074 | 1 | 27700074 | _ | _ | _ |
| HSW | TBL | M1 M2 | 1:27912648 | 1 | 27912648 | _ | _ | _ |
| HSW | TBL | M1 M2 | 1:28175274 | 1 | 28175274 | _ | _ | _ |
| HSW | TBL | M1 M2 | 1:28566634 | 1 | 28566634 | _ | _ | _ |
| HSW | TBL | M1 M2 | 1:29069982 | 1 | 29069982 | _ | _ | _ |
| HSW | TBL | M1 M2 | 1:29243562 | 1 | 29243562 | _ | _ | _ |
| HSW | TBL | M1 M2 | 1:29379166 | 1 | 29379166 | Ca_22562 | Ca1:29379105..29381000 | Putative pentatricopeptide repeat-containing protein |
| HSW | TBL | M1 M2 | 1:29579998 | 1 | 29579998 | _ | _ | _ |
| HSW | TBL | M1 M2 | 1:29939706 | 1 | 29939706 | _ | _ | _ |
| HSW | TBL | M1 M2 | 1:30124685 | 1 | 30124685 | _ | _ | _ |
| HSW | TBL | M1 M2 | 1:30235486 | 1 | 30235486 | _ | _ | _ |
| HSW | TBL | M1 M2 | 1:30397449 | 1 | 30397449 | _ | _ | _ |
| HSW | TBL | M1 M2 | 1:30882040 | 1 | 30882040 | _ | _ | _ |
| HSW | TBL | M1 M2 | 1:31004002 | 1 | 31004002 | _ | _ | _ |
| HSW | TBL | M1 M2 | 1:31094655 | 1 | 31094655 | _ | _ | _ |
| HSW | TBL | M1 M2 | 1:31173355 | 1 | 31173355 | _ | _ | _ |
| HSW | TBL | M1 M2 | 1:31282074 | 1 | 31282074 | _ | _ | _ |
| HSW | TBL | M1 M2 | 1:31346707 | 1 | 31346707 | _ | _ | _ |
| HSW | TBL | M1 M2 | 1:31433071 | 1 | 31433071 | _ | _ | _ |
| HSW | TBL | M1 M2 | 1:31507015 | 1 | 31507015 | _ | _ | _ |
| HSW | TBL | M1 M2 | 1:31667537 | 1 | 31667537 | _ | _ | _ |
| HSW | TBL | M1 M2 | 1:32081265 | 1 | 32081265 | _ | _ | _ |
| HSW | TBL | M1 M2 | 1:32195371 | 1 | 32195371 | _ | _ | _ |
| HSW | TBL | M1 M2 | 1:32346709 | 1 | 32346709 | _ | _ | _ |
| HSW | TBL | M1 M2 | 1:32869041 | 1 | 32869041 | _ | _ | _ |
| HSW | TBL | M1 M2 | 1:32990748 | 1 | 32990748 | _ | _ | _ |
| HSW | TBL | M1 M2 | 1:33354318 | 1 | 33354318 | Ca_19441 | Ca1:33323295..33369645 | Probable galacturonosyltransferase 11 |
| HSW | TBL | M1 M2 | 1:33428414 | 1 | 33428414 | _ | _ | _ |
| HSW | TBL | M1 M2 | 1:33462887 | 1 | 33462887 | _ | _ | _ |
| HSW | TBL | M1 M2 | 1:33614613 | 1 | 33614613 | _ | _ | _ |
| HSW | TBL | M1 M2 | 1:33759538 | 1 | 33759538 | _ | _ | _ |
| HSW | TBL | M1 M2 | 1:33935474 | 1 | 33935474 | _ | _ | _ |
| HSW | TBL | M1 M2 | 1:34167008 | 1 | 34167008 | _ | _ | _ |
| HSW | TBL | M1 M2 | 1:34218461 | 1 | 34218461 | _ | _ | _ |
| HSW | TBL | M1 M2 | 1:34895908 | 1 | 34895908 | _ | _ | _ |
| HSW | TBL | M1 M2 | 1:34960479 | 1 | 34960479 | _ | _ | _ |
| HSW | TBL | M1 M2 | 1:35046092 | 1 | 35046092 | _ | _ | _ |
| HSW | TBL | M1 M2 | 1:35077378 | 1 | 35077378 | _ | _ | _ |
| HSW | TBL | M1 M2 | 1:35097234 | 1 | 35097234 | _ | _ | _ |
| HSW | TBL | M1 M2 | 1:35124857 | 1 | 35124857 | _ | _ | _ |
| HSW | TBL | M1 M2 | 1:38665088 | 1 | 38665088 | _ | _ | _ |
| HSW | TBL | M1 M2 | 1:38707076 | 1 | 38707076 | _ | _ | _ |
| HSW | TBL | M1 M2 | 1:38748348 | 1 | 38748348 | _ | _ | _ |
| HSW | TBL | M1 M2 | 1:40199659 | 1 | 40199659 | _ | _ | _ |
| HSW | TBL | M1 M2 | 1:42131076 | 1 | 42131076 | _ | _ | _ |
| HSW | TBL | M1 M2 | 1:42499537 | 1 | 42499537 | _ | _ | _ |
| HSW | TBL | M1 M2 M5 | 1:4712062 | 1 | 4712062 | _ | _ | _ |
| HSW | TBL | M1 M2 M5 | 1:4719871 | 1 | 4719871 | Ca_00555 | Ca1:4715466..4724391 | Probable prolyl 4-hydroxylase 4 |
| HSW | TBL | M1 M2 M5 | 1:4724009 | 1 | 4724009 | Ca_00555 | Ca1:4715466..4724391 | Probable prolyl 4-hydroxylase 4 |
| HSW | TBL | M1 M2 M5 | 1:4737857 | 1 | 4737857 | _ | _ | _ |
| HSW | TBL | M1 M2 M5 | 1:4743995 | 1 | 4743995 | _ | _ | _ |
| HSW | TBL | M1 M2 M5 | 1:4768558 | 1 | 4768558 | Ca_00561 | Ca1:4767537..4769629 | Putative uncharacterized protein |
| HSW | TBL | M1 M2 M5 | 1:4772934 | 1 | 4772934 | _ | _ | _ |
| HSW | TBL | M1 M2 M5 | 1:4780345 | 1 | 4780345 | Ca_00562 | Ca1:4779836..4783224 | Putative E3 ubiquitin-protein ligase RF298 |
| HSW | TBL | M1 M2 | 2:35876038 | 2 | 35876038 | Ca_09763 | Ca2:35874908..35878711 | Aspartic proteinase-like protein 2 |
| HSW | TBL | M1 M2 M5 | 2:35893060 | 2 | 35893060 | _ | _ | _ |
| HSW | TBL | M1 M2 M5 | 4:12342257 | 4 | 12342257 | _ | _ | _ |
| HSW | TBL | M1 M2 M5 M6 | 4:12525550 | 4 | 12525550 | Ca_04475 | Ca4:12522142..12526684 | Transmembrane ascorbate ferrireductase 1 |
| HSW | TBL | M1 M2 M5 | 4:12795793 | 4 | 12795793 | _ | _ | _ |
| HSW | TBL | M1 M2 M5 | 4:12798952 | 4 | 12798952 | _ | _ | _ |
| HSW | TBL | M1 M2 M5 | 4:12802488 | 4 | 12802488 | _ | _ | _ |
| HSW | TBL | M1 M2 M5 | 4:12926117 | 4 | 12926117 | _ | _ | _ |
| HSW | TBL | M1 M2 M5 | 4:13184656 | 4 | 13184656 | _ | _ | _ |
| HSW | TBL | M1 M2 M5 | 4:13214301 | 4 | 13214301 | _ | _ | _ |
| HSW | TBL | M1 M2 M5 | 4:13331455 | 4 | 13331455 | Ca_04558 | Ca4:13328534..13333733 | Protein TIFY 4B |
| HSW | TBL | M1 M2 M5 | 4:13381856 | 4 | 13381856 | _ | _ | _ |
| HSW | TBL | M1 M2 M5 | 4:13418361 | 4 | 13418361 | _ | _ | _ |
| HSW | TBL | M1 M2 M5 | 4:13720217 | 4 | 13720217 | _ | _ | _ |
| HSW | TBL | M1 M2 | 6:2529519 | 6 | 2529519 | _ | _ | _ |
| HSW | TBL | M1 M2 M5 | 6:2529763 | 6 | 2529763 | _ | _ | _ |
| HSW | TBL | M1 M2 M4 M5 | 6:2533425 | 6 | 2533425 | _ | _ | _ |
| HSW | TBL | M1 M2 M5 | 7:32895545 | 7 | 32895545 | _ | _ | _ |
| HSW | TBL | M1 M2 M5 | 7:33013532 | 7 | 33013532 | _ | _ | _ |
| HSW | TBL | M1 M2 M5 | 7:33015473 | 7 | 33015473 | _ | _ | _ |
| HSW | TBL | M1 M2 M5 | 7:33016980 | 7 | 33016980 | _ | _ | _ |
